# Bench-to-bedside development of multifunctional flexible embolic agents

**DOI:** 10.7150/thno.80213

**Published:** 2023-04-01

**Authors:** Dawei Wang, Wei Rao

**Affiliations:** 1 Key Lab of Cryogenics, Technical Institute of Physics and Chemistry, Chinese Academy of Sciences, Beijing, 100190, China; 2 Beijing Key Lab of CryoBiomedical Engineering, Technical Institute of Physics and Chemistry, Chinese Academy of Sciences, Beijing, 100190, China; 3 School of Future Technology, University of Chinese Academy of Sciences, Beijing 100049, China

**Keywords:** Hepatocellular carcinoma (HCC), Transarterial chemoembolization (TACE), Micro/nano materials, Particulate embolic agents, Ga based liquid metal, Multifunctional flexible embolic agents

## Abstract

Transarterial chemoembolization (TACE) has been demonstrated to provide a survival benefit for patients with unresectable hepatocellular carcinoma (HCC). However, conventional TACE still faces limitations associated with complications, side effects, unsatisfactory tumor responses, repeated treatment, and narrow indications. For further improvement of TACE, additional beneficial functions such as degradability, drug-loading and releasing properties, detectability, targetability, and multiple therapeutic modalities were introduced. The purpose here is to provide a comprehensive overview of current and emerging particulate embolization technology with respect to materials. Therefore, this review systematically identified and described typical features, various functions, and practical applications of recently emerging micro/nano materials as particulate embolic agents for TACE. Besides, new insights into the liquid metals-based multifunctional and flexible embolic agents were highlighted. The current development routes and future outlooks of these micro/nano embolic materials were also presented to promote advancement in the field.

## 1. Introduction

Hepatocellular carcinoma (HCC) is one of the most frequently diagnosed primary liver cancer and is becoming one of the leading causes of cancer-related mortality because of complex tumor pathogenesis, disease recurrence, and metastases 1-3. Despite significant advances in screening, diagnosis, and treatment, patients with HCC still face a poor prognosis with a 5-year survival rate of approximately 10% to 15% 2, 4. This could be interpreted by the fact that although the surveillance programs of at-risk populations are widely implemented, most patients with HCC are first diagnosed at an intermediate or advanced stage (Barcelona Clinic Liver Cancer stage B or C) 2, 5 when curative treatments such as surgical excision, liver transplantation and percutaneous ablation (radiofrequency ablation, microwave ablation, etc.) cannot be applied 6-14. Therefore, the preferred treatment for patients with advanced-stage HCC is topical or palliative treatment rather than curative treatment. Recently, the importance of interventional treatment is increasing for patients with advanced-stage HCC 15, because of superior features such as substantially reduced therapeutic complication, minimally invasive, delaying disease progression and corresponding improved life quality. Among them, transarterial chemoembolization (TACE) is one of the most critical palliative treatment options for inoperable HCC 8, 16-19, which can also be implemented as a preoperative adjuvant treatment for patients with resectable HCC 15, 20.

**Figure 1:**
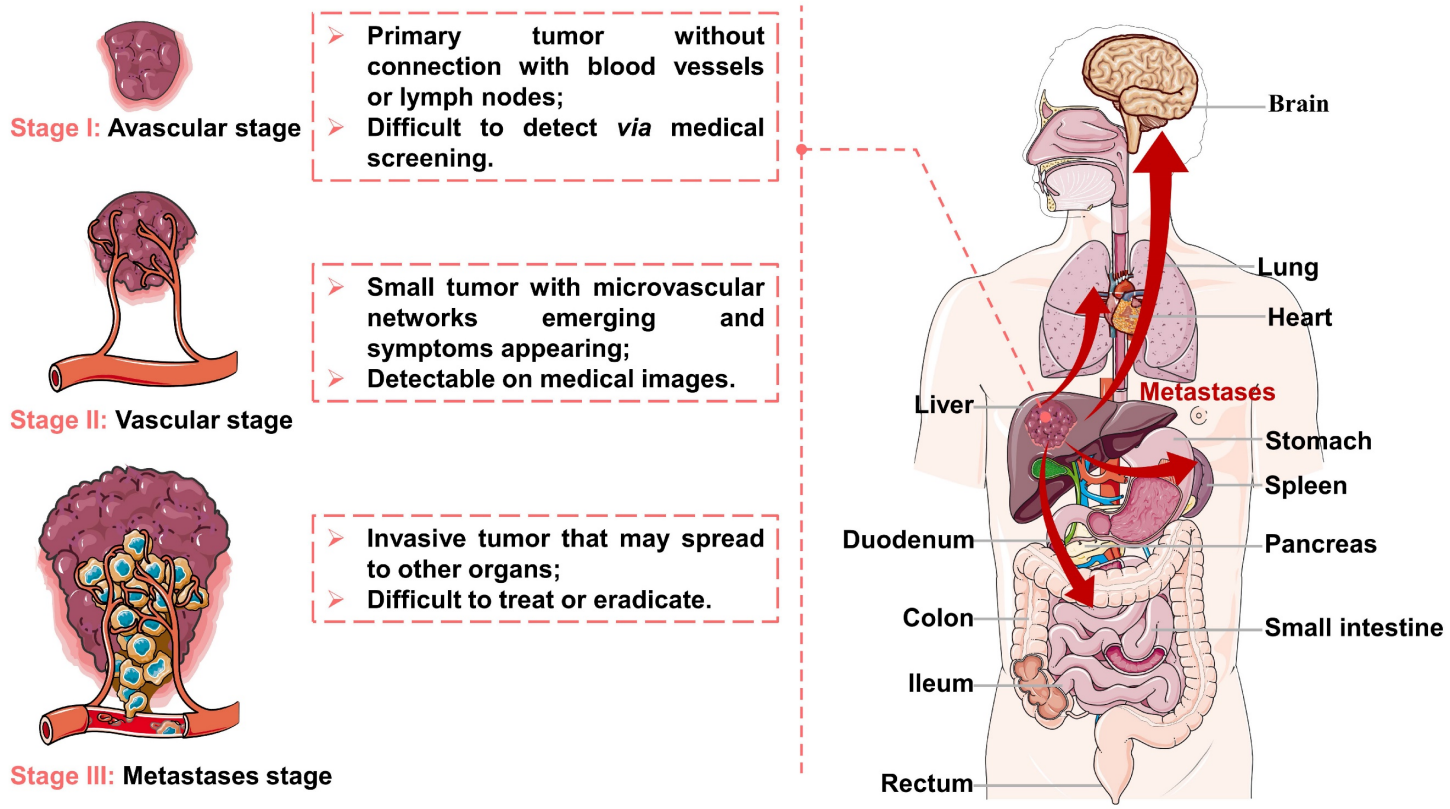
** Representation of the liver cancer stages: (I) avascular stage; (II) vascular stage; (III) metastases stage.** Created in Smart.Servier.com.

Viral infections, genetic disorders, chemical toxins, and metabolic syndrome are the main factors contributing to the development of HCC, which can modulate oxidative stress, cancer stem cells, hypoxia, hormonal system, inflammatory/immune system and epithelial-mesenchymal transition 21-23. After tumorigenesis, the growth and metastasis of invasive tumors depend on tumor angiogenesis 24, 25, and their evolution can be divided into three stages (as illustrated in Figure 1). First, a tumor appears in the liver and starts to grow by obtaining nutrients from its immediate environment. When growing to a certain size, the tumor will secrete proangiogenic factors to accelerate neovascularization (referred to as angiogenesis) due to ischemia and a decrease of pH in local tissues, thus providing oxygen and nutrients required for tumor growth. Finally, the tumor becomes invasive while the tumor cells may spread to other organs. Because of the unique evolutionary process of HCC, TACE can inhibit tumor growth and metastasis by cutting off the pathways of oxygen, nutrient, and tumor cell transmission.

**Figure 2:**
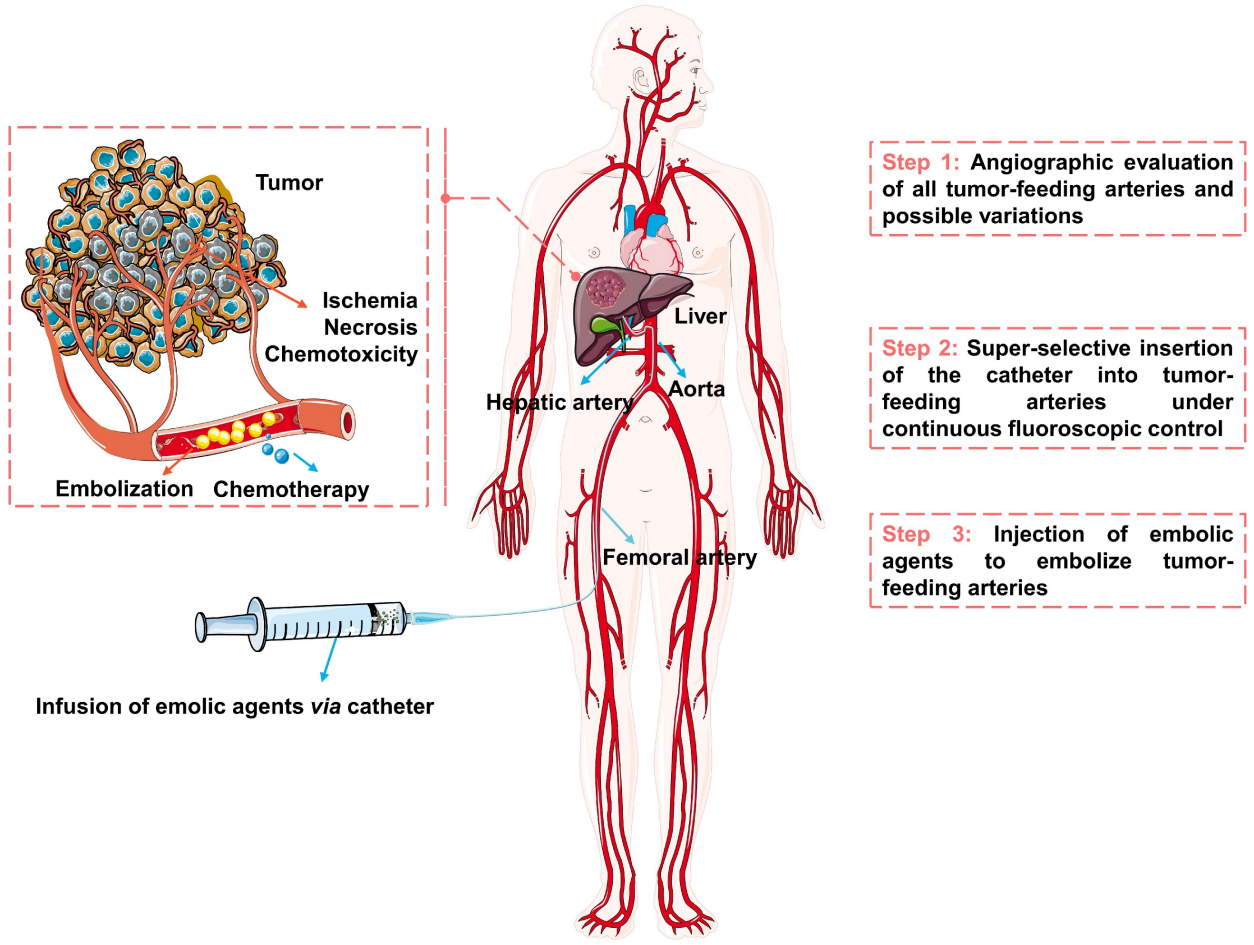
**The schematic diagram of transarterial embolization for hepatocellular carcinoma.** Created in Smart.Servier.com.

The schematic diagram of the TACE procedure was shown in Figure 2. Briefly, the embolic materials and chemotherapeutic agents are selectively injected into the targeted tumor vessels under the guidance of precise imaging assistance, then the supply of nutrients and oxygen is cut off and the chemotherapeutic drugs are released, ultimately causing ischemia, necrosis, and chemo-toxicity to the tumor cells 26-29. TACE can treat HCC without affecting normal hepatic parenchyma, because of the difference in blood supply between hypervascular tumors (mainly from the hepatic artery 30, 31) and normal liver tissue (unique dual blood supply, namely: two-thirds of the necessary blood supply comes from the portal vein and one-third from hepatic artery 26). However, not every patient with HCC may benefit from TACE. Considering tumor size, tumor location, extrahepatic spread, underlying liver function, and patient status, etc 12, the best candidates who may benefit from TACE are patients with asymptomatic lesions and preserved liver function without extrahepatic spread or vascular invasion 32. The optimum treatments need to be carefully selected for the individual patient, such as blank transarterial embolization (TAE), conventional transarterial chemoembolization (cTACE), TACE with drug-eluting beads (DEB-TACE), transarterial radioembolization (TARE), arterial embolization hyperthermia (AEH) and other treatments in combination with TACE. Transcatheter vascular occlusion can be achieved by using multiplex forms of embolic agents, including devices (e.g., coils, stents, and balloons), liquids (e.g., glue and *in situ* gelling solution), sclerosing agents (e.g., alcohol and thrombin) and particulates (e.g., polymer and hydrogels particulates) 33, 34. Among them, particulates are becoming ideal embolic agents for integrated and tailored transarterial embolization therapy, due to their versatile functionality 35.

**Figure 3:**
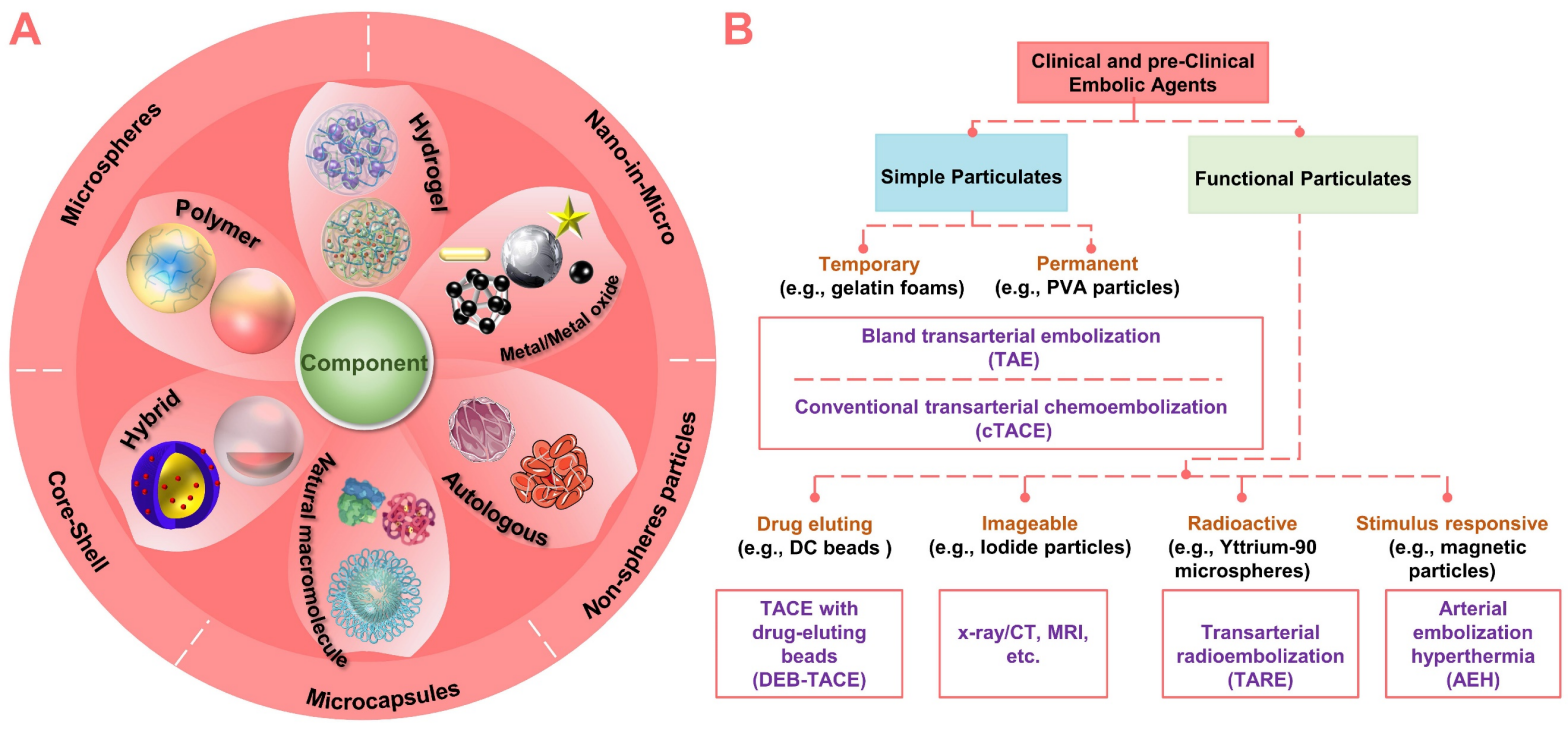
** Summary of clinical and pre-clinical particulate embolic agents.** (A) Structure and component of micro/nano embolic agents. (B) Classification of micro/nano embolic agents.

Nowadays, with the innovation of micro/nano technology and material design, more and more micro/nano embolic particulates (materials: polymeric, metallic, natural, or composite; structure: non-spherical, spherical, porous, capsule, hollow or shell-core type) are emerging (Figure 3A). Since the innovation of materials will eventually exert the diagnostic and therapeutic functions of embolic agents. Therefore, this review systematically identified and described recently emerging micro/nano materials as particulate embolic agents for TACE, with emphasis on materials, typical features, various functions, and practical applications (Figure 3B). The content began with a description of the basic embolic matrix, followed by a detailed introduction of the typical embolic microspheres used clinically (e.g., drug-eluting beads, imageable embolic microspheres, and radioactive microspheres). Then, the vision was expanded to the stimuli-sensitive embolic microspheres in terms of properties, performances, and underlying application (e.g., combination therapy of TACE with various interventional thermal/nonthermal ablation modalities). After that, new insights into the liquid metals-based multifunctional flexible embolic agents were presented, which are expected to have a broader impact on future angiography and intravascular embolization. Finally, the current development routes and future outlooks of these emerging micro/nano embolic materials were also concluded.

## 2. Micro/nano materials used for particulate embolic agents

### 2.1 Simple embolic agents for TAE or cTACE

Conventional transarterial chemoembolization TACE (cTACE) typically involves the sequential infusion of chemotherapeutic agents mixed with Lipiodol and embolic agents into the tumor-feeding hepatic artery branch, while blank transarterial embolization (TAE) aims to achieve devascularization in absence of chemotherapeutic agents 15, 19, 36-39. The embolic agents implanted in the tumor-feeding vessels provide a single but fundamental function of embolization. Simple embolic agents include: (1) biodegradable embolic agents for temporary embolization, e.g., albumin, gelatin, starch, dextran, chitosan, alginate, carboxymethyl cellulose, polylactic-co-glycolic acid (PLGA), polyanhydride, and polyesters, etc. 40, and (2) nonbiodegradable embolic agents for permanent embolization, e.g., polyvinyl alcohol (PVA) particles, trisacryl gelatin particles, and derivative particles. The common feature of these basic embolic agents is their good biocompatibility, and they are often used as matrix materials to prepare various functional embolic agents, especially those currently under investigation.

#### 2.1.1 Temporary gelatin-based embolic agents

Autologous blood clots were the first developed temporary embolic materials for endovascular embolization, after that, autologous subcutaneous muscle and tissue were also used as embolic materials 41, 42. These naturally autologous embolic materials are nontoxic and biocompatible because they can be individualized for each patient 42. However, they are rarely used in current clinical due to the increasing clinical demand and the rapid advancements in particle embolization technology. The currently available gelatin-based embolic agents (various forms: gelatin foam, gelatin powder, gelatin microspheres, etc.) are derived from purified porcine gelatin (non-antigenic carbohydrate), which can be enzymatically digested for temporary embolization. The gelatin-based embolic agents mechanically embolize tumor arteries to block or retard blood flow, while the internal reticular structure can enhance the embolization effect by promoting thrombus formation (reticulating the red blood cells and platelets). Gelatin foams are one of the common embolic agents 16, 43, which have been introduced for TAE in the late 1970s 44, 45. Gelatin foams are often marketed as Gelfoam (e.g., Pharmacia & Upjohn Co., New York), and are available in different configurations (e.g., sponges and sterile sheets) with typical particle sizes range of 0.5~2 mm 46. During embolization, the injectable slurry is formed through the physical mixing of the gelatin foams with the iodinated contrast media (for radiopacity) 36, 47. Additionally, gelatin foams as hemostatic embolic agents can promote blood clotting and reduce blood loss, and its porous structure can be a scaffold to promote cell adhesion and tissue regeneration 34. Millimeter-scaled gelatin foams are likely to clump in larger arteries, and thus cannot penetrate the smaller vessels in distal tissues. Although micron-sized gelatin powder (size ranging from 40 to 60 μm 34) can reach smaller vessels to achieve more distal vessel obstruction, it is more likely to cause insufficient or non-targeted embolization 48-50. Furthermore, gelatin microspheres with regular shapes and variable diameters (ranging from 40 to 1200 µm) have also been produced, such as: gelatin sponge microparticles (GSMs) 51, and gelatin microparticles (GMPs) 29. The gelatin microspheres with accurate particle size can effectively avoid ectopic embolization, which is crucial for localized, targeted, and tailored embolization. Due to the degradable nature of the gelatin matrix, the vessel recanalization may occur within a few weeks, showing advantages in hepatic TAE/cTACE and facilitating repeated intra-arterial treatment 29. However, uncontrolled degradation kinetics of these gelatin embolic particles may cause uncertainties in clinical trials, thus hindering their widespread application.

#### 2.1.2 Permanent PVA-based embolic agents

Polyvinyl alcohol (PVA)-based particles are mature polymeric embolic agents with good safety, long-term biocompatibility, and non-biodegradability, which can be used for permanent or semi-permanent embolization. The initially used PVA particles were obtained by mechanically fragmenting, screening, and separating the PVA polymer blocks 52, which were used as vascular embolic agents in clinical practice since the 1970s 53. When injected into tumor-feeding arteries, the PVA particles can also achieve devascularization followed by thrombus formation. Different from temporary embolic agents, the recanalization in PVA particles-mediated TAE/cTACE may occur over several months due to particle migration and vascular remodeling 54. However, due to irregular shapes, inhomogeneous sizes 55, and charged/hydrophobic surfaces 34, the PVA particles are prone to aggregate and thus may lead to catheter occlusion 56 or accidental blockage of the proximal larger vessels 57. The main challenge is the unpredictable embolic behavior caused by the dimension uncertainty between the PVA particles and the target tumor-feeding arteries 34. For this reason, several vendors have developed PVA-based microspheres with regular shapes and dimensional accuracy. For example, spherical PVA microspheres (Contours SE, Boston Scientific, Natick, MA), PVA microspheres crosslinked with acrylic polymer (Bead Block, Biocompatibles UK, Surrey), and PVA microspheres with hydrogel core (LC Bead, Biocompatibles Inc. and RITA, Manchester, GA), which are commercially available with sizes ranges of 100-300, 300-500, 500-700, and 700-900 μm 58. With structural optimization and surface modification, these PVA-based microspheres may overcome some of the disadvantages related to PVA particles, providing more precise treatment control.

### 2.2 Drug-eluting beads for DEB-TACE

During the cTACE, although the dual efficacy of chemotherapy and embolization can be achieved, there are still shortcomings associated with complications, side effects, and unsatisfactory treatment responses, such as insufficient or nontarget embolization 59, uncontrolled and unsustainable chemotherapeutic drug release 19, 60, ineffective drugs concentration in tumor tissue 60, and high incidence of liver-related systemic toxicities 17, 38, 61, etc. The newer drug-eluting beads (DEBs)-mediated TACE (DEB-TACE) utilizes a single delivery system of DEBs 62, which can reduce the concentration of chemotherapeutic drugs in systemic circulation and maintain effective drug concentration within the target tumor for a prolonged period 60, 63, showing less systemic side effects 64 and more benefits over cTACE according to clinical studies 36, 39. In particular, the TACE procedure could also be significantly simplified since the DEBs play a dual role, acting as both an embolic agent and a drug carrier 65. So far, a variety of DEBs or also called drug-eluting microspheres (DEMs) have been developed, and some are commercially available (Table 1). And there are also many anti-tumor drugs available for TACE (such as doxorubicin, epirubicin, idarubicin, mitoxantrone, carboplatin, cisplatin, oxaliplatin, 5-fluorouracil, gemcitabine, mitomycin C, and paclitaxel, etc. 66), among which doxorubicin is one of the most commonly used in clinic 67. It should be noted that under TACE-induced ischemic stress, retention of hypoxia-inducible factor 1 (HIF-1), upregulation of angiogenic receptors, and increased nuclear proliferation rates may occur within embolized tumor tissue 68. Among them, HIF-1 as a controlling factor may regulate the subsequent release of multiple angiogenic factors (such as vascular endothelial growth factor, insulin-like growth factor and basic fibroblast growth factor), thereby inducing angiogenesis 21, 69. Fortunately, anti-angiogenic drugs (e.g., sorafenib) could also be combined with DEB-TACE to inhibit the induction of HIF-1, while favorable tumor inhibition rate has been demonstrated in phase-II trials for patients with unresectable HCC 70. The matrix of DEBs is a critical determinant of the drug loading and releasing mechanisms 71, while the microstructure (e.g., porosity) is another factor that modulates drug loading and releasing profiles 72. Thus, we will discuss the typical DEBs mainly based on the drug loading and releasing mechanisms.

#### 2.2.1 Drug-eluting beads based on ion-exchange mechanism

**Figure 4:**
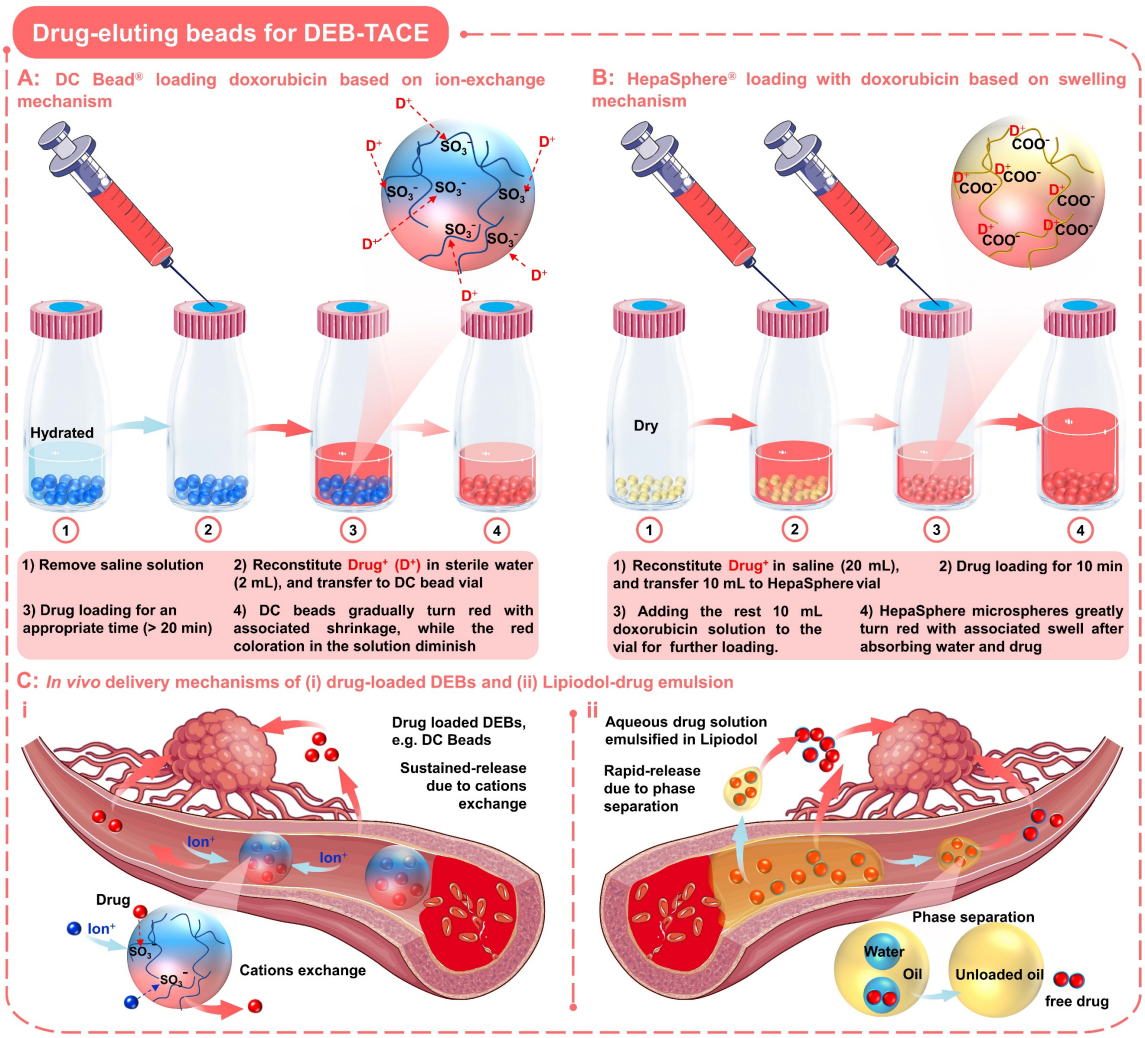
** Drug-eluting beads for DEB-TACE.** Schematic diagram of drug loading process and mechanism of two commonly used DEBs: (A) DC Bead loading with doxorubicin based on ion-exchange mechanism; (B) HepaSphere loading with doxorubicin based on swelling mechanism. (C) *In vivo* delivery mechanisms of (i) drug-loaded DEBs and (ii) Lipiodol-drug emulsion. Created with BioRender.com and Smart.Servier.com.

This type of negatively charged DEBs are capable of loading and eluting positively charged chemotherapeutic agents *via* an ion-exchange mechanism (Figure 4A) 18. Among them, the commercially available DC Beads are the most commonly used and well-characterized in DEB-TACE for HCC 75, 76. DC beads are typically available with size ranges of 100-300, 300-500, 500-700, and 700-900 µm (Table 1), and are generally supplied in the hydrated form (in saline solution with appropriate ionic strength). In particular, DC Beads are PVA-based microspheres containing anionic sulfonate groups that allow the sequestering of positively charged drugs, such as doxorubicin, and irinotecan, *via* Coulomb charge interactions 26.

In order to load the drugs into the DC beads, it is necessary to remove the saline solution before TACE treatment, and then mixed with the drug solution for an appropriate time (as the loading process outlined in Figure 4A) 65. When loading with doxorubicin (red), the DC beads will gradually turn red with associated shrinkage, while the red coloration in the solution will diminish (Figure 4A). Up to 99% of doxorubicin uptake occurs between 20 min and 24 h, depending on loading concentration and bead size. In addition, DC beads also show advantages in drug elution performance compared to traditional Lipiodol-doxorubicin emulsions (Figure 4C), sustained-release Vs. rapid-release). The underlying mechanisms may be that the elution kinetics of DC beads are primarily governed by the ionic environment and surface area, since ions need to penetrate the surface and diffuse into the hydrogel matrix to displace the drugs from the sulfonate moieties (Figure 4C, i) 65, 77, 78. In comparison, the water-soluble drugs will be rapidly released from the Lipiodol-doxorubicin emulsions, as the Lipiodol droplets will rapidly separate from the emulsion (Figure 4C, ii) 65, 77. Furthermore, the clinical researches have shown that the high drug-loading efficacy and targeted/sustained drug-releasing properties of DC beads did contribute to improved response (higher rates of complete response, objective response, and disease control) and tolerability (significant reduction in severe liver toxicity and significantly lower rate of doxorubicin-related side effects) compared to cTACE in HCC patients 76, 79. However, the DC beads with the negatively charged surface can only load cationic drugs and the non-biodegradable nature may also lead to late-stage inflammatory responses due to persistent occlusion 80.

#### 2.2.2 Drug-eluting beads based on swelling mechanism

In order to expand the types of drugs that can be loaded by DEBs, the absorption properties of the gel materials have been developed, so that anionic drugs can be loaded *via* a swelling mechanism (Figure 4B). As a typical example, HepaSphere microspheres are hydrophilic, superabsorbent polymer microspheres, which can be bound with doxorubicin, irinotecan, epirubicin, cisplatin or oxaliplatin 81. HepaSphere microspheres are available in the 'dry state' with size ranges of 50-100, 100-150, and 150-200 µm (Table 1). When exposed to aqueous-based media, the dry HepaSphere microspheres may undergo dramatic morphological changes after absorbing liquid (e.g., becoming soft, deformable, and accompanied by volume expansion (up to 64 × volume 32)). Due to the high degree of compliance and flexibility, the hydrated HepaSphere microspheres can be easily delivered through the currently available microcatheters 26, and can match the shape of tortuous and narrow vessels for a more complete embolization 81, 82. In contrast with DC Beads, the HepaSphere microspheres also possess a negative ionic charge, which allows the binding of cationic drug molecules *via* electrostatic interactions (Figure 4B) 81-83. However, due to the porous structure of the HepaSphere microspheres, the chemotherapeutic drugs are bound throughout the volume, while the DC Beads are on the surface (Figure 4A-B) 81, 83. A comparative study by Jordan O et al. has shown that the doxorubicin loading efficiency and releasing profile of HepaSphere microspheres (400-600 µm) was similar to that of DC Beads (500-700 µm) 83. They observed incomplete release of doxorubicin in saline (release rate over 1 week: 27 ± 2 % for DC beads and 18 ± 7 % for HepaSphere microspheres; P = 0.013), which may explain the low systemic exposure and suboptimal anticancer function of doxorubicin 81, 83. Besides, they also observed some fractured HepaSphere microspheres after drug release 83. In short, the HepaSphere microspheres are also an appropriate option in DEB-TACE for HCC, showing favorable safety and efficacy 84, 85.

In addition, other DEBs based on swelling mechanisms have also been developed, such as gelatin 86, poly (lacticco-glycolic acid) (PLGA) 87 and alginate 88 based polymeric hydrogels microspheres 89. These absorbent microspheres seem to be feasible for drug loading, especially for those anionic (e.g., ketoprofen) 86. Furthermore, the insoluble drugs can also be directly encapsulated into the embolic particles, while the drug release is associated with structure destruction.

### 2.3 Imageable embolic microspheres

The visualization of embolic agents is critical in interventional therapy, which may facilitate the effective localization of embolic agents, improve therapy control and follow-up assessment, and increase the success rate of TACE. However, conventional embolic microspheres and drug-eluting beads still face the drawback of lacking real-time tracking capabilities, which brings difficulties for physicians to precisely target the treatment area, appropriately detect and standardize end points 90, 91, opportunely identify potential insufficient or non-target embolization 59, thereby compromising clinical safety and outcomes. The visualization capabilities can be obtained by simple physical mixing of the embolic agents with the iodinated contrast media 36, 47. Unfortunately, the fluidity and diffusibility of the contrast medium may cause systemic toxicity and misdiagnosis 65, 92, 93. Therefore, there has always been widespread interest in the development of intrinsically imageable embolic microspheres. Initially, researchers aimed to incorporate as many radiopaque components as possible in the embolic microspheres to improve their X-ray imaging capabilities. Later, as medical imaging technology evolved, a wealth of medical diagnostic techniques was applied to TACE. Thus, the focus shifted to developing multiple imaging materials to render embolic agents more comprehensive imaging modalities. In general, the basic concept remains unchanged, while it is more desirable to find the delicate balance between the need for sufficient imageability and the maintenance of appropriate physicochemical properties 38.

Currently, diverse inclusions (e.g., iodine-containing species, heavy elements, superparamagnetic substances, and phase-changing materials) have been investigated to impart embolic particle imaging capabilities (Table 2).

### 2.3.1 X-ray Visible embolic microspheres

**Figure 5:**
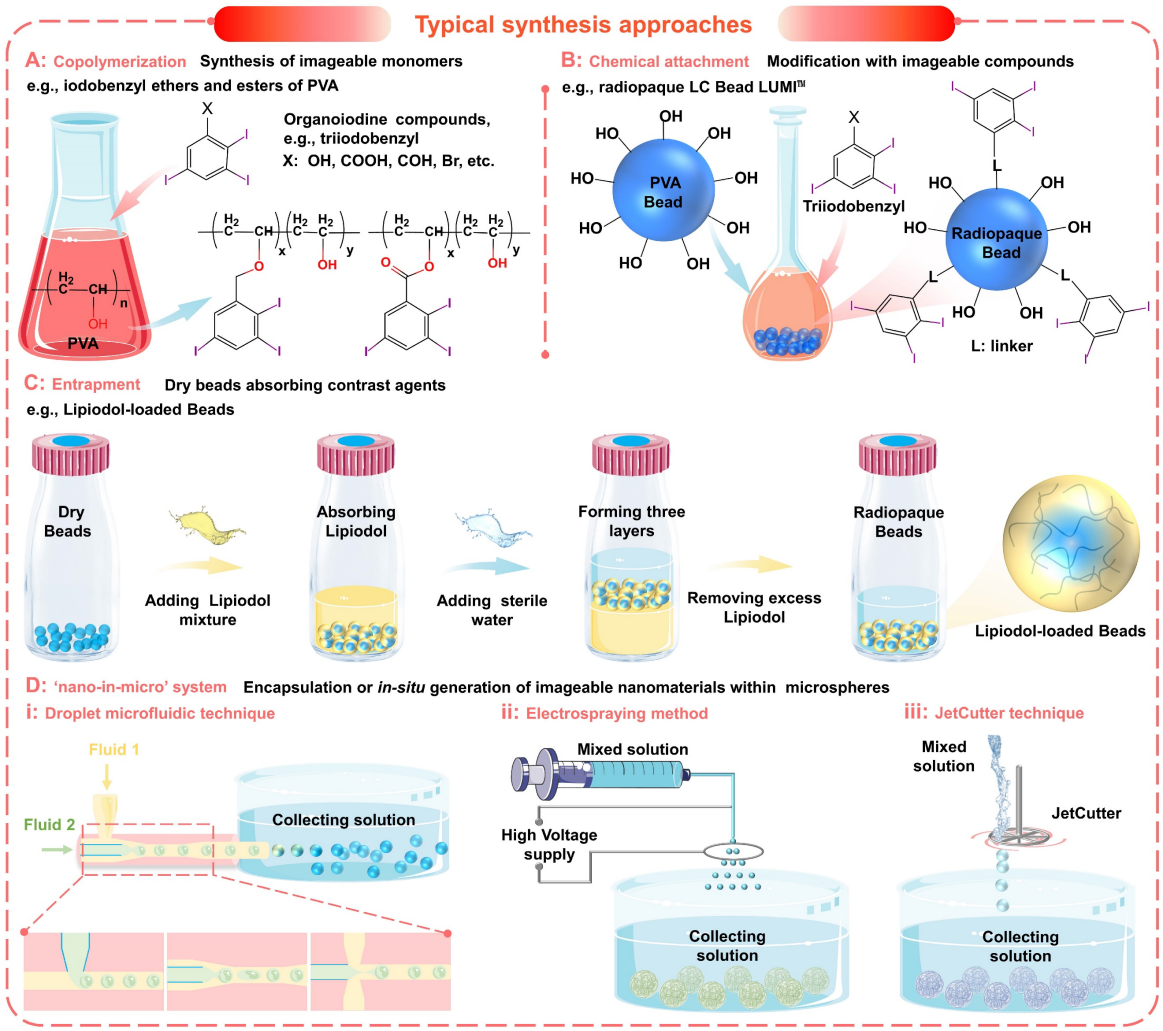
** Typical synthesis approaches of imageable embolic microspheres.** (A) Synthesis of imageable monomers by copolymerization, e.g., iodobenzyl ethers and esters of poly (vinyl alcohol) (PVA). (B) Chemical attachment of imageable compounds onto the prefabricated particles, e.g., radiopaque LC Bead LUMI^TM^. (C) Entrapment of contrast agents within the host particle structure, e.g., Lipiodol-loaded Beads. (D) Encapsulation or *in situ* generation of imageable nanomaterials within microspheres to form a 'nano-in-micro' (or called 'nano-on-micro') system: (i) droplet microfluidic technique; (ii) electrospraying method; (iii) JetCutter technique.

**Figure 6:**
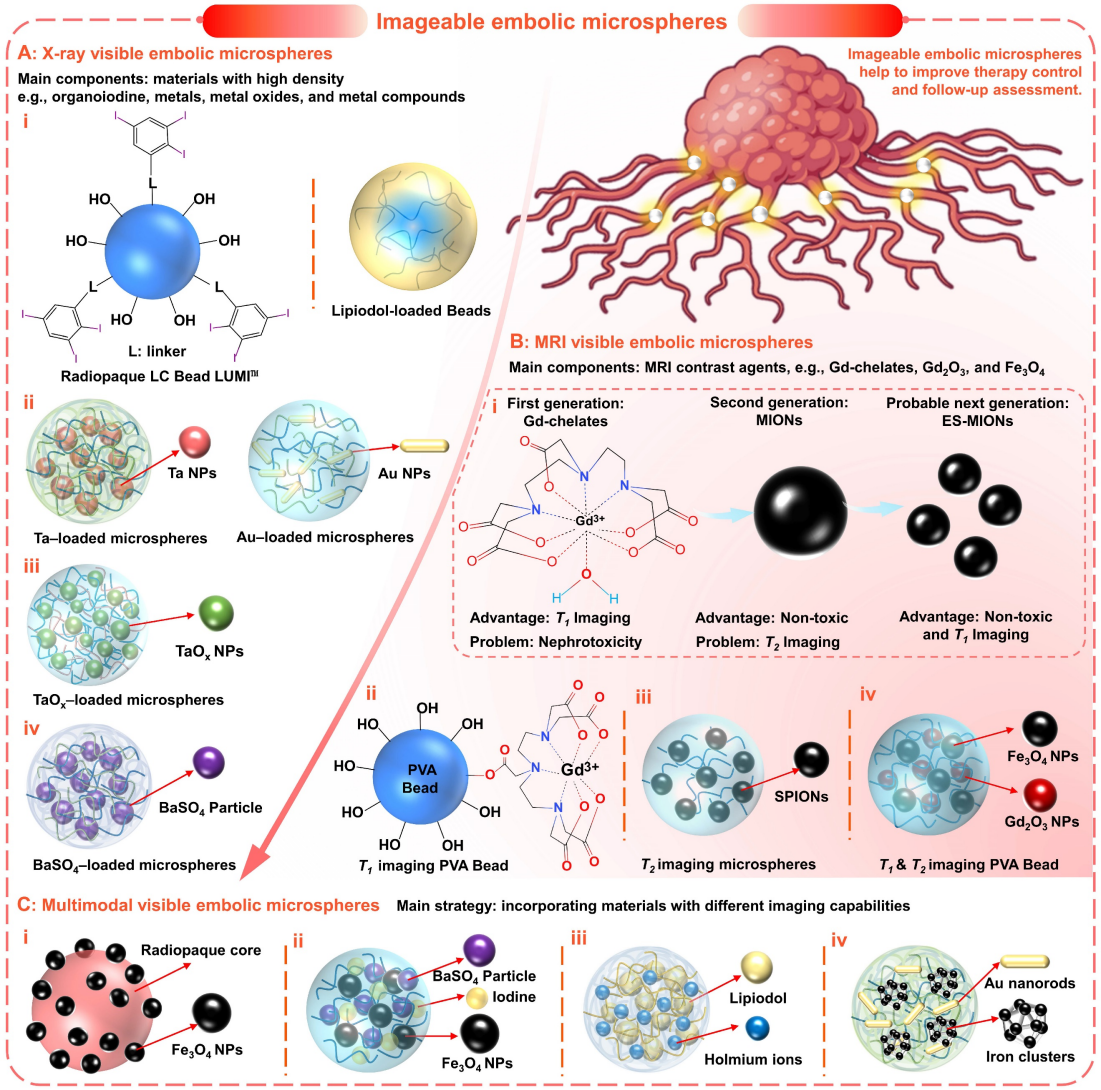
** Imageable embolic microspheres help to improve therapy control and follow-up assessment.** (A) Typical X-ray visible embolic microspheres based on radiopaque materials, such as (i) organoiodine (e.g., triiodobenzyl, Lipiodol), (ii) metals (e.g., tantalum (Ta), gold (Au)), (iii) metal oxides (e.g., TaO_x_), and (iv) metal compounds (e.g., BaSO_4_). (B) Typical MRI visible embolic microspheres based on MRI contrast agents: (i) main problems and advantages of current and future MRI contrast agents, e.g., T_1_-weighted contrast agents (gadolinium (Gd) chelate), T_2_-weighted contrast agents (magnetic iron oxide nanoparticles, MIONs), and T_1_-weighted contrast agents (extremely small MIONs, ES-MIONs, smaller than 5 nm); (ii) PVA Bead modified with Gd (III) chelates for T_1_ imaging; (iii) superparamagnetic iron oxide nanoparticles (SPIONs, ~12nm) loaded polymerized microspheres for T_2_ imaging; (iv) PVA hybrid microspheres loaded with Gd_2_O_3_ and Fe_3_O_4_ nanoparticles (~5 nm) for T_1_ & T_2_ imaging. (C) Multimodal (X-ray and MRI) visible embolic microspheres were realized by incorporating materials with different imaging capabilities: (i) macroparticle consist of iodine-containing core and Fe_3_O_4_ particles coating; (ii) Embozene microspheres modified with iodine impregnation and BaSO_4_/Fe_3_O_4_ precipitation; (iii) hydrated alginate microspheres loaded with holmium and Lipiodol; (iv) alginate microspheres containing radiopaque gold nanorods and magnetic iron clusters. Created with BioRender.com.

X-ray-based imaging (including fluoroscopy (i.e., digital radiography), computed tomography (CT), and digital subtraction angiography (DSA)) has shown significant advantages due to high diagnosis efficiency, high image resolution, and accurate diagnosis results 121. In general, elements with high atomic numbers and atomic mass all have the potential to absorb X-rays. Therefore, radiopaque properties are often imparted by introducing materials with high densities, such as organoiodine, metals, metal oxides, and metal compounds (Figure 5 and Figure 6A).

The incorporation of organoiodine compounds (such as iodine species containing iodinated benzyl groups (Figure 5A-C and Figure 6A, i) has been widely studied for the introduction of intrinsic radiopacity into polymeric microspheres 38. The major approaches include a) copolymerization (Figure 5A) of radiopaque monomers with embolic matrix, b) chemical attachment (Figure 5B) of radiopaque moiety on the prefabricated particles, and c) entrapment (Figure 5C, no bonding) of iodine-containing compounds within the host particle structure 102, 104, 119. The first two approaches provide synthetic flexibility and allow the incorporation of high iodine content (even as high as 70%, Table 2) to improve radiopacity. The chemical reaction will bring a certain degree of adverse effects on the necessary properties (e.g., hydrophilicity, softness, smoothness, flexibility, uniformity, and dispersibility) 38. However, if the iodine content is appropriately balanced (25~30wt%), these microspheres can still effectively embolize the hepatic arteries and maintain fine visibility. Besides, it is worth noting that there are some commercially available radiopaque drug-eluting beads (e.g., LC Bead LUMI™, DC Bead LUMI™, and X-Spheres) have overcome some of these issues (Table 3) 73, 122. Moreover, the radiopaque microspheres can also be prepared by loading commercial X-ray contrast agents (e.g., Lipiodol), benefiting from the absorbent properties of dry microspheres (as illustrated schematically in Figure 5C) 62. These Lipiodol-loaded beads show intrinsic radiopacity, superior embolization ability (sufficient embolization, adjustable embolization diameter, and less ectopic embolism compared to pure Lipiodol) 38, and maintain sustained drug release characteristics 116. However, since no bond is formed, it is important that the organoiodine compounds remain entrapped and do not overflow over time 38. In general, the incorporation of organoiodine compounds is a practical and efficient approach to impart inherent radiopacity and will show broad prospects in future clinical evaluation.

To satisfy the precise imaging requirement, another innovative strategy is proposed to incorporate functional nanomaterials into microspheres to form a 'nano-in-micro' (or called 'nano-on-micro') system (Figure 5D and Figure 6A, ii) 71, 123. There are two major approaches: a) encapsulation and b) *in situ* generation. For most metal nanoparticles or metal oxide particles that are difficult to synthesize (such as Ta (Figure 6A, ii) 93 and TaO_x_ (Figure 6A, iii) 103 nanoparticles), they can be encapsulated into the embolic matrix. For some nanoparticles that can be synthesized by precipitation or redox reactions (such as BaSO_4_ (Figure 6A, iv) 88, 112, Bi_2_S_3_ 124, Au (Figure 6A, ii) 125), they can be *in situ* generated within the embolic structure.

As a typical example, microfluidic technology can be conveniently used to realize 'nano-in-micro' structured alginate embolic microspheres, since alginate can quickly cross-link with metal ions to form a hydrogel (Figure 5D and Figure 6A, ii-iv). For example, Yang's group applied a one-step electrospraying method (Figure 5D, ii) to synthesize tantalum nanoparticles (Ta NPs) loaded calcium alginate microspheres (Ta@CaAlg) (Figure 6A, ii), by spraying a mixture of Ta NPs and sodium alginate into CaCl_2_ solution 93. In this study, renal embolization was performed in rabbits using optimized Ta@CaAlg microspheres (330 μm, containing 10 wt% Ta NPs). And the relative X-ray signal intensity was 6490, which is comparable to that of Iodixanol solution (7355). Besides, the *in vivo* results showed that Ta@CaAlg microspheres possessed both embolic and contrast agent properties. Compared with radiolucent calcium alginate microspheres (without tantalum), Ta@CaAlg microspheres were visible in both digital radiography and CT scans 4 weeks after embolization, indicating the potential for real-time imaging and long-term assessment. Similarly, Yang's group also used the electrospraying method (Figure 5D, ii) to fabricate barium alginate microspheres loaded with *in situ* synthesized BaSO_4_ particles (BaSO_4_@BaAlg microspheres) (Figure 6A, iv), by spraying a mixed solution (sodium alginate and Na_2_SO_4_ mixture) into the collecting bath (BaCl_2_ solution) 88. Monodispersed BaSO_4_@BaAlg microspheres with sizes ranging from 200 to 1800 µm could be achieved by varying the electrospray parameters. While the X-ray visibility was demonstrated through *in vitro* test (CT values: 7 wt % BaSO_4_, 2172 ± 164 HU Vs. Iohexol solution containing 300 mg iodine (I) mL^-1^, 3703 ± 153 HU), and the radiodensity remained stable for over 52 days. Through routine renal artery embolization, the embolic effect and intrinsic radiopacity of the BaSO_4_@BaAlg microspheres were confirmed. However, more studies on the *in vivo* distribution and metabolism mechanisms are needed to better understand how these chemically stable and water-insoluble inclusions are cleared.

#### 2.3.2 MRI Visible embolic microspheres

Recently, magnetic resonance imaging (MRI) has also received extensive attention in TACE therapy, considering no ionizing radiation, multi-azimuth imaging, plentiful diagnostic information, high resolution for soft tissue, and 3D visualization/localization 123, 126-128. With these benefits, there is a great incentive to transition to MRI-guided interventional therapy. Currently, some examples of real-time MRI applications are routinely performed or will be available in the near future, such as real-time MRI guided cardiovascular interventional therapy 128-131, thermal ablation 132, cryoablation 133-136, and radiotherapy 137, 138, etc. While MRI technologies continue to evolve, perhaps the most pressing focus is on translation to clinical TACE.

MRI technology is a clinical imaging modality that measures the nuclear magnetic resonance (NMR) signals emitted by protons in human bodies under a magnetic field 139. There are two types of MRI contrast agents that can significantly improve MRI performance by affecting the MR signal properties of the surrounding tissues 140. a) T_1_-weighted contrast agents (or positive contrast agents), which can shorten the longitudinal relaxation times (T_1_) of protons, leading to brighter images in T_1_-weighted MRI; b) T_2_-weighted contrast agents (or negative contrast agents), which can shorten the transverse relaxation times (T_2_) of protons, resulting in darker images in T_2_-weighted MRI. Currently, most commercially available MRI contrast agents are T_1_-weighted contrast agents based on gadolinium (Gd) chelates (Figure 6B, i) 139, 140. However, the U.S. Food and Drug Administration (FDA) has recommended a prohibition in all patients with acute renal insufficiency 141, due to the Gd-associated nephrotoxicity 139. Therefore, MRI contrast agents based on magnetic iron oxide nanoparticles (MIONs) have received increasing attention because of their superior biocompatibility and safety (iron is an essential element in the human body) (Figure 6B, i) 139. In general, superparamagnetic iron oxide nanoparticles (SPIO NPs) are commonly served as T_2_-weighted contrast agents, such as FDA approved Feraheme (vascular imaging) and Feridex I.V. (liver and spleen imaging) 142. But the dark images caused by T_2_-weighted contrast agents may be confused with signals from other pathogens, thus affecting the diagnosis. Fortunately, extremely small MIONs (ES-MIONs, smaller than 5 nm) have recently emerged as potential positive contrast agents (Figure 6B, i) 139.

Previously, there have been sporadic attempts to combine Gd (III) chelate with embolic microspheres to realize MRI visualization. For instance, Cilliers et al. modified the Gd (III) chelate on the surface of commercial PVA particles (Contour^®^, diameter 45-150 µm) with Gd (III) content of 45.5 µg/mg (chemical attachment: Figure 5B and Figure 6B, ii) 107. After modification, the T_1_ relaxation times decreased by more than 80 %, from 1200 ms to 225 ms. In another study, van Elk et al. encapsulated temperature-sensitive liposomes (loaded Gd (III) chelate) into hydrogel microspheres (325 µm), to monitor drug release and microgel deposition (JetCutter technique: Figure 5D, iii) 109. After mild hyperthermia, the Gd (III) chelates were almost completely released and the T_1_-weighted MRI signal intensity increased more than 3 times. Although these studies indicated that the Gd (III) chelates were marginally released or controllable released *in vitro*, they still cannot eliminate the possibility of nephrotoxicity since these chelates may form strong complexes with biological ligands *in vivo* 143, 144. Therefore, the development of MIONs-based embolic microspheres with better biocompatibility has rekindled the interest of scholars 67, 123, 145-148. For example, Li et al. synthesized SPIONs-loaded polymeric microspheres (SPMs, 100-300, 300-500 500-700, 700-900 µm) by inverse suspension polymerization method (Figure 6B, iii) 147. Due to the size limitation, SPMs can only be applied to negative contrast enhancement of T_2_-weighted MRI, which is difficult to correctly display the position and distribution of microspheres *in vivo*. In contrast, Wang et al. prepared PVA hybrid microspheres with dual-modal MRI imageability, in which *in situ* synthesized Gd_2_O_3_ and Fe_3_O_4_ nanoparticles act as T_1_ and T_2_-weighted MRI contrast agents, respectively (droplet microfluidic technique: Figure 5D, i and Figure 6B, iv) 123. This is a compromise approach to obtain T_1_/T_2_-weighted MRI imageability, and perhaps embolic microspheres based entirely on MIONs and ES-MIONs will be developed in the near future. Overall, although X-ray-based imaging is the dominant technology for real-time monitoring during the current TACE procedure, the MRI-guided TACE under development will show advantages in the future 149.

#### 2.3.3 Multimodal Visible embolic microspheres

In addition, the embolic microspheres that integrated multimodal imaging capabilities may cope with the needs of different diagnostic scenarios and provide more comprehensive information. The most universal and practical strategy is to incorporate materials with different imaging capabilities into the embolic matrix (Figure 6C). Years ago, H. Bartling et al. proposed the formation of multimodal-visible embolic macroparticles (diameter, 40-200 µm) by suspension homopolymerization of glycidyl methacrylate with 2-methacryloyloxyethyl (2,3,5-triiodobenzoate), which consist of X-ray visible iodine-containing core and MRI-visible Fe_3_O_4_ particles coating (150 nm) (Figure 6C, i) 113. The *in vivo* renal embolization revealed that at least partial devascularization was achieved, thus confirming the embolic efficiency. And the signal changes caused by these particles were found in the three imaging modalities (DSA, CT, and MRI), which contribute to monitoring the* in vivo* localization and distribution of the particles. Besides, Stampfl et al. modified commercial Embozene microspheres (polyphosphazene-coated poly (methyl methacrylate), Boston Scientific, Marlborough, MA, USA) by impregnation of iodine and precipitation of barium sulfate and iron oxide, to achieve multimodal imaging visibility for radiography, MR imaging, and CT (Figure 6C, ii) 114. Moreover, hydrated holmium-Lipiodol-alginate microspheres (Ho-lip-ams, 570 ± 12 µm) were prepared by cross-linking alginate-oil emulsion (1:1, alginate: oil, w/w) with chloride salt of holmium (HoCl_3_, 25mM) (JetCutter technique: Figure 5D, iii and Figure 6C, iii) 111. Within the microspheres, the inclusion of Lipiodol offered visualization capabilities for fluoroscopy and CT, while the holmium ions (0.38 ± 0.01% (w/w)) allowed MR imaging. When incubated in fetal calf serum (FCS) at 37 ℃, these microspheres remained intact, but Lipiodol was gradually released for two weeks. When these microspheres were injected into the organ, a similar deposition pattern was also observed in different imaging modalities. Additionally, Kim et al. demonstrated that the inclusion of radiopaque gold nanorods and magnetic iron clusters in the alginate microspheres could also achieve MRI/CT dual-modality visualization (Figure 6C, iv) 150. As an alternative CT contrast agent, gold NPs may improve the limitations of conventional iodine contrast agents 151. In short, utilizing complementary characteristics of X-ray-based imaging and MRI, the TACE procedure can be optimized (guiding intra- and post-procedural visualization, preventing ectopic embolization, accurately determining the endpoint of embolization, and improving follow-up examinations) 111, 113. However, it will undoubtedly increase the complexity of the embolic system and raise concerns about stability, toxicity, and biocompatibility, despite the feasibility of the incorporation method. Thus, researchers should also consider reasonable trade-offs between complex embolic systems and multiple multimodal imaging capabilities.

### 2.4 Microspheres for radioembolization

Historically, radiotherapy has played an important role in cancer treatment, which is independent of chemical therapy or other energy-based ablation techniques 152. Due to the radiosensitive nature of normal hepatic tissue (< 30 Gy 153), tumoricidal radiation dose (> 70 Gy 154) to solid liver tumors will lead to severe complications (including liver failure and other fatal gastrointestinal symptoms 155), which limits the application in HCC. Thus, selective delivery of radioactive embolic microspheres into the tumor arterial is essential for safe and successful radiotherapy in hepatic malignancies 152. Consequently, the concept of transarterial radioembolization (TARE, or as selective internal radiation therapy (SIRT)) was introduced, which is a combination of brachytherapy and embolization using the same technical principle as cTACE 15, 156.

**Figure 7:**
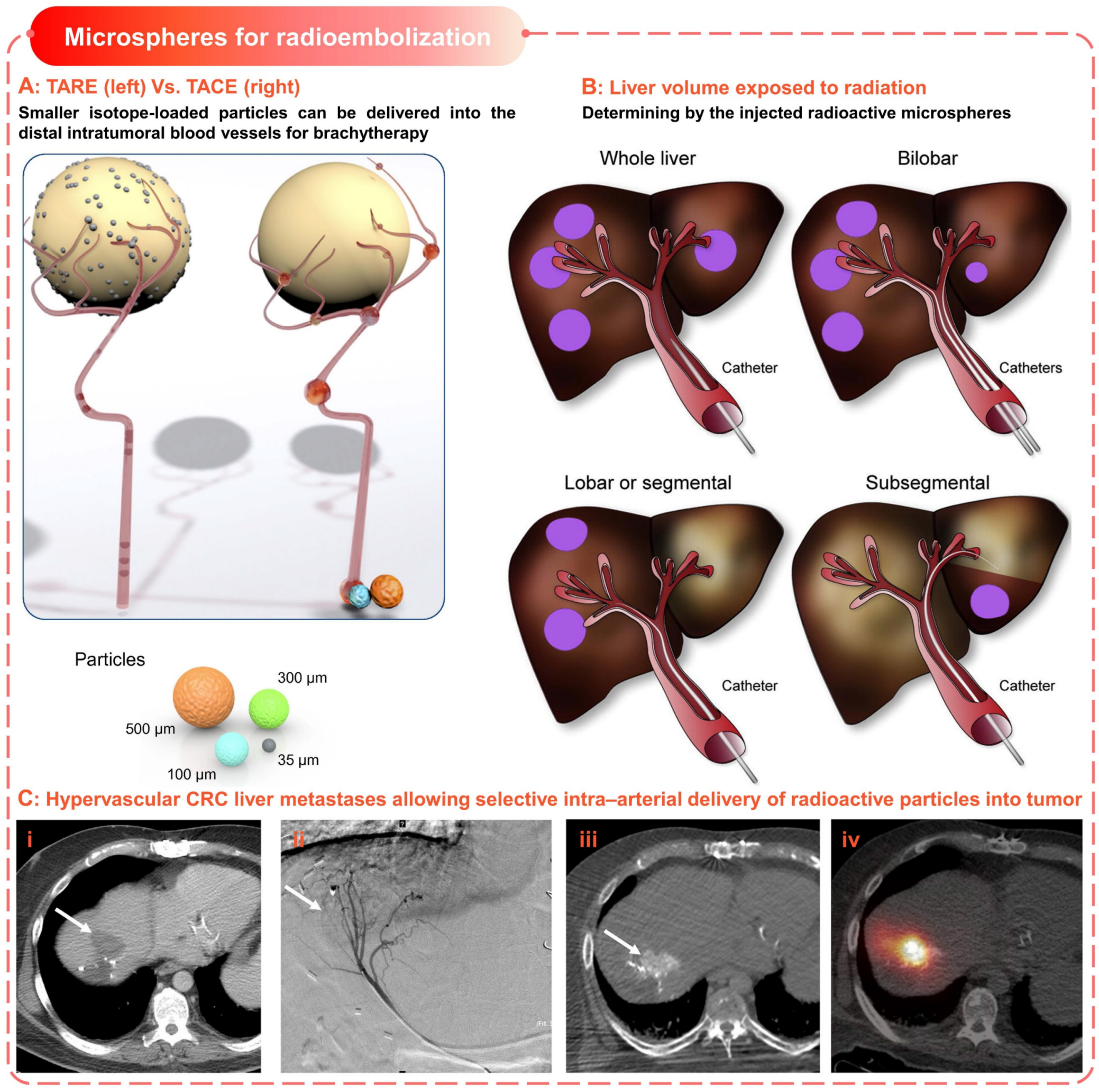
** Microspheres for radioembolization.** (A) Schematic illustrates the difference of devices and procedures used for TARE and TACE; Smaller isotope-loaded particles can be delivered into the distal intratumoral blood vessels for brachytherapy. Adapted with permission from 168, Copyright 2012 Elsevier. (B) Liver volume exposed to radiation is defined by the injected radioactive microspheres. Adapted with permission from 168, Copyright 2012 Elsevier. (C) Hypervascular colorectal cancer (CRC)liver metastases allowing selective intra-arterial delivery of radioactive particles into the tumor. (i) CRC liver metastases (arrow), (ii) catheter angiogram shows arterial blood supply, (iii) hypervascularity of lesion, (iv) bremsstrahlung SPECT/CT after injection of ^90^Y microspheres. Adapted with permission from 169, Copyright 2017 Society of Nuclear Medicine.

The therapeutic effect of TARE is mainly exerted by the carried radiation source rather than the chemical or ischemic effect (Figure 7A-B), for which microspheres loaded with Yttrium-90 (^90^Y) are commonly used 156. Yttrium-90 is a pure β emitter with a short half-life (64.2 h) and limited tissue penetration (average 2.5 mm, maximum 11 mm) 156, which allows local high-dose radiation while minimizing the risk of radiation-induced normal hepatic necrosis (Figure 7C). There are two types of commercially available ^90^Y microspheres for treating hepatic neoplasms, namely 1) TheraSphere^®^ (BTG International, London, United Kingdom) and 2) Sir-Spheres^®^ (Sirtex Medical Europe, Bonn, G) 152, 156. All other radioactive microspheres are either investigational or not clinical, such as ^166^Ho and ^188^Re-based microspheres 157, 158. TheraSphere^®^ is composed of nonbiodegradable glass, in which ^90^Y radioisotope act as an integral constituent of the glass matrix.

And it was approved in 1999 for radiotherapy or as neoadjuvant therapy before transplantation, suitable for patients with unresectable HCC in whom the hepatic arterial catheters can be appropriately positioned. Besides, SirSpheres^®^ consists of biodegradable resin-based microspheres with ^90^Y radioisotopes attached to the surface. And it received premarket approval in 2002 for the treatment of patients with colorectal liver metastases (CRLM) in conjunction with intrahepatic Floxuridine. Other features of these two microspheres, such as specific gravity, radioactivity, and radiation dose, were detailed in Table 4. These different features could explain the differences in hypothetical applications, administration modes, and activity dose for each patient 156, but there were no significant differences in clinical efficacy 159. In early clinical trials, the TARE was only used as a salvage option after the failure of first-line therapy 152. However, the subsequent phase I and phase II clinical investigation 160 and several retrospective studies 15, 161, 162 have revealed the safety and efficacy of TARE using^ 90^Y isotope-loaded particles in the treatment of HCC. Specifically, the HCC patients treated with TARE showed similar therapy responses compared to alternative therapies including cTACE, TACE, and chemotherapy (sorafenib) 163-166. In general, TARE is becoming a promising treatment option for unresectable primary or secondary liver malignancies 152, 167.

### 2.5 Microspheres for Arterial Embolization Hyperthermia

**Figure 8:**
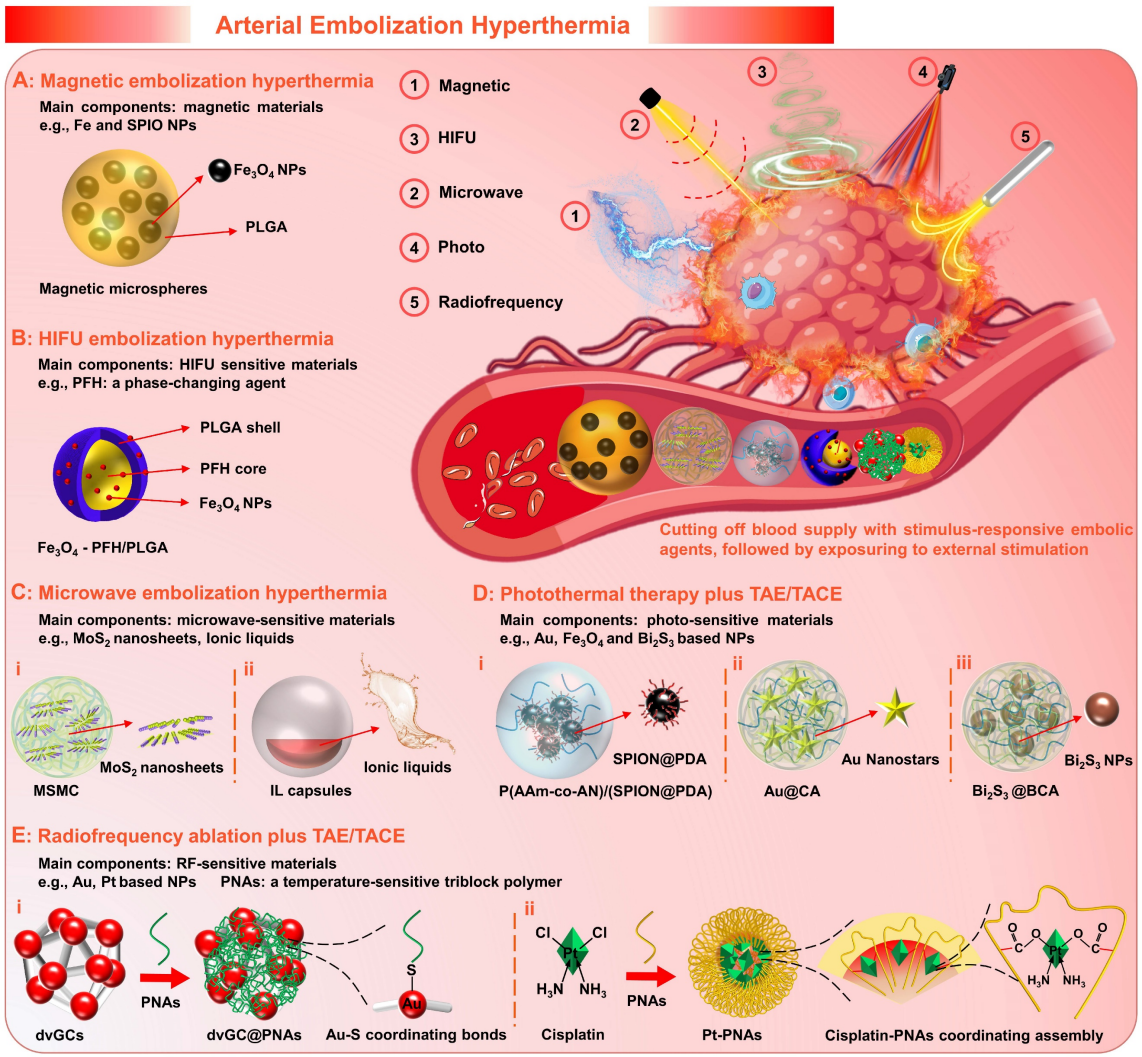
** Arterial embolization hyperthermia is hopeful to develop a complementary treatment model, which can not only introduce supplemental therapeutic mechanisms for TACE therapy, but also reduce the blood flow “cooling” effect for hyperthermia (schematic illustration).** (A) Magnetic embolization hyperthermia based on magnetic microspheres, e.g., poly (lactic-co-glycolic acid)(PLGA)-magnetic microspheres. (B) High-intensity focused ultrasound (HIFU) sensitive micro/nano materials for HIFU embolization hyperthermia, e.g., Fe_3_O_4_ nanoparticles-integrated PLGA capsules with phase-changing agents (perfluorohexane (PFH)) core (Fe_3_O_4_-PFH/PLGA). (C) Microwave (MW) sensitive micro/nano materials for MW embolization hyperthermia, e.g., (i) alginate microspheres embedded with molybdenum sulfide nanosheets (MSMC), (ii) MW susceptible ionic liquids (IL) loaded micro/nanocapsules. (D) Photo-sensitive micro/nano materials for TAE/TACE plus photothermal therapy (PTT), e.g., (i) poly(acrylamide-co-acrylonitrile) microspheres encapsulated with polydopamine coated superparamagnetic iron oxide nanoparticles (P(AAm-co-AN)/(SPION@PDA)), (ii) calcium alginate (CA) hydrogel microspheres containing Au nanostars (Au@CA), (iii) alginate microspheres encapsulated with bismuth sulfide nanoparticles (Bi_2_S_3_@BCA). (E) Radiofrequency (RF) sensitive embolic agents for coordinating TAE/TACE and radiofrequency ablation, e.g., (i) RF-sensitive dual-valent gold nanoclusters (dvGC) modified with temperature-sensitive poly (N-isopropylamide-co-acrylic acid) (PNAs), (ii) cisplatin-crosslinking PNA nanogels (Pt-PNAs). Created with BioRender.com.

Generally, as a palliative treatment, TACE cannot eliminate viable tumor cells, which may result in the failure of TACE due to local tumor recurrence and distant metastasis 120, 170. For the development and further applications of the TACE, supplemental therapeutic mechanisms, including magnetic induction hyperthermia (MIH) 145, 171, microwave ablation (MWA) 172, 173, high-intensity focused ultrasound (HIFU) thermotherapy 120, 170, 174, photothermal therapy (PTT) 175, 176, and radiofrequency ablation (RFA) 177, 178, etc., have been introduced to enhance the therapeutic effects. Extensive clinical studies have confirmed improved outcomes through combination therapy of TACE with interventional thermal/nonthermal ablation modalities 14, 179-181. In addition to the sequential implementation approaches, the use of stimulus-responsive embolic agents is also a feasible combination method that can enhance the efficacy of targeted hyperthermia, simplify the surgical procedure and minimize the invasiveness. Therefore, the experimental concept of arterial embolization hyperthermia (AEH) has been proposed (Figure 8). Its basic principle is to selectively embolize the hepatic tumor supply arteries with stimulus-responsive embolic agents, followed by external stimulation exposure to generate hysteretic heating of the embolized particles and hence the surrounding tissues 182. For this purpose, micro/nano materials that are sensitive to external energy fields are often incorporated into the embolic matrices, such as magnetic-sensitive nanoparticles 171, microwave-sensitive nanosheets 101, HIFU-sensitive nanocapsules 120, photothermal-sensitive nanostars 125, and radiofrequency-sensitive nanoclusters 183, etc. Besides, it can also realize active drug release *via* external energy regulation, which will enable a more flexible dosing regimen. Moreover, it is worth mentioning that supplemental thermotherapy can effectively enhance the tumoricidal effects of TAE/TACE, while intravascular embolization can also promote the local accumulation of heat, thus is hopeful to develop a mutually reinforcing treatment model.

#### 2.5.1 Magnetic embolic microspheres

Magnetic embolization hyperthermia is a branch of magnetic targeted hyperthermia, that utilizes magnetic particles to achieve selective arterial embolization and heat generation (magnetic hysteresis loss effects or Neel relaxation under alternating magnetic field (AMF)) 184. For magnetic embolization hyperthermia, iron and superparamagnetic iron oxide nanoparticles (SPIO NPs) are the main components of the magnetic induction mediator within embolic microspheres. Because they possess a set of required properties, e.g., non-toxic, non-immunogenic, biocompatible, biodegradable, high magnetization saturation, and radiopacity 185. For instance: Liang et al. fabricated poly (lactic-co-glycolic acid) (PLGA)-magnetic microspheres (MMs, controllable size 100 to 1000 μm) embedded with Fe_3_O_4_ NPs (20 nm) by a rotating membrane emulsification system (Figure 8A) 171. And, the MMs were successfully employed for combining TAE and magnetic ablation (TAEMA) to treat orthotopic VX2 liver tumors-bearing rabbits. In this study, the designed magnetic field and microsphere parameters (number, size) can directly adjust the required temperature rise, thereby leading to tumor cell apoptosis (42~46 °C, hyperthermia) or necrosis (>50 °C, ablation). The *in vitro* and *in vivo* results showed that the tumor edge could be heated to more than 15 °C within 30 min when exposed to AMF (390 kHz, 12 A or 500 kHz, 16 A) after TAE, while the temperature increase near the normal liver parenchyma was negligible (< 5 °C). Besides, the phase transition of PLGA (from glass state to rubber state) was also observed when heated to the glass transition temperature (Tg, 50 °C), which may cause adhesion and aggregation to enhance the embolic effect. Theirs follow-up research confirmed that the MMs could effectively load drugs (drug loading coefficient of 8.1 %, and encapsulation efficiency of 94.6 %), and also revealed that the temperature plays a decisive role in drug release 186. In addition, the simulation and experimental studies of Qiu et al. have shown that the magnetic embolic microspheres can be controllably aggregated in the presence of a magnetic field, showing the potential for the magnetic field-controlled embolization 145. Compared to direct injection hyperthermia (DIH) with magnetic fluid perfusion (uneven distribution and poor retention in the tumor tissue), magnetic embolization hyperthermia can achieve durable and repeatable targeted magnetic hyperthermia 182. And several studies have confirmed its preliminary feasibility and efficacy in animal models 182, 187-191.

Simply put, magnetic embolization hyperthermia is a typical paradigm for combining targeted hyperthermia with TAE or TACE, providing a feasible synergistic treatment strategy. However, it is worth considering that long-term exposure to high-intensity AMF may cause patient discomfort, although the application of AMF with frequencies of 0.05-1.5 MHz is a safe method to minimize the impact on healthy tissues.

#### 2.5.2 HIFU-sensitive embolic particles

High-intensity focused ultrasound (HIFU) hyperthermia is an extracorporeal and non-invasive technique that induces coagulative necrosis by thermal injury and mechanical stress 120, 192. Its combination with TACE is another experimental technique undergoing preclinical evaluation in addition to magnetic embolization hyperthermia 170, 184. Since several retrospective studies have demonstrated that the combination therapy of TACE plus HIFU ablation was more beneficial than monotherapy (e.g., TACE, chemotherapy) in different patients with HCC (e.g., children, adults, and elderly; with primary, unresectable, or metastatic liver cancer), which could improve the prognosis and prolong the life expectancy without increasing the incidence of adverse reactions 174, 193-195.

Recently, there have been several studies on HIFU-sensitive micro/nano materials for HIFU embolization hyperthermia. For instance, You et al. explored the HIFU-sensitive composite nanocapsules (Fe_3_O_4_-PFH/PLGA) with SPIO NPs-integrated PLGA capsules and phase-changing agents (perfluorohexane (PFH)) core by a double-emulsion process, which can be used for the synergistic treatment of TACE plus HIFU ablation (Figure 8B) 120. During the *in vivo* experiment, the TACE was performed on the VX2 liver tumor-bearing rabbits *via* transarterial injection of a mixture of Fe_3_O_4_-PFH/PLGA and Lipiodol emulsion, and the HIFU ablation was implemented (0.8 MHz, focal lengths 135 to 155 mm, power 180 W, and exposure duration 5 s) after TACE. In particular, the accumulation and retention of micro/nanocapsules within the targeted areas were enhanced by the TACE, thereby increasing the energy deposition and enlarging the tumor coagulation volume. Besides, the bubbles were generated due to the temperature-induced phase transformation of the PFH core, which further strengthen the embolization and HIFU ablation effects. In addition, this micro/nano system can be applied for ultrasound, magnetic resonance, and photoacoustic tri-modality imaging due to the integration of several functional materials, which is beneficial to assist tumor localization and prognostic diagnosis. Although many questions remain to be verified, such as whether the freely moving bubbles will cause ectopic embolization, it is undeniable that this innovative research may stimulate broad interest in the development of HIFU-sensitive embolic agents.

#### 2.5.3 Microwave sensitive embolic microspheres

Microwave ablation (MWA) utilizes electromagnetic energy to induce tumor coagulative necrosis, which offers the advantages of short delivery time, and a large/predictable ablation zone 14. Early studies have examined its safety and efficiency as an effective treatment for liver cancer 172, 173, 196. And several randomized controlled trials (RCT) have demonstrated that the combination therapy of TACE plus MWA is more effective than TACE monotherapy, in respect to higher tumor necrosis rate, better tumor response, longer tumor progression time, and lower complication rate 197-199.

In recent years, microwave (MW)-sensitive micro/nano materials have been developed for MW embolization hyperthermia, which can synergistically maximize tumor necrosis by the targeted hyperthermia and the reduced blood flow “cooling” effect. For example, Meng's group prepared MW-sensitive embolic microspheres by embedding molybdenum sulfide nanosheets within alginate microspheres (MSMCs, 5.6 ± 1.8 μm) (Figure 8C, i) 101. In the *in vivo* experiments, the MSMCs were injected into the VX2 liver tumor-bearing rabbits *via* a transcatheter arterial route, achieving well distribution in the marginal and internal regions of the tumors. Under MW irradiation, the temperature at the tumor site rapidly increased to 50 °C within 1 min and reached approximately 60 °C within 5 min. Such high and persistent hyperthermia could cause protein denaturation to completely kill cancer cells. Besides, after 3 days of treatment, the ablation zone was observed to be 5 times larger than that of the MWA alone. Thus, this study validated that the combination therapy of MWA and TAE/TACE relying on MW-sensitive embolic microspheres is a promising option for large tumors. In addition, Meng's group also developed chemical drugs and MW-susceptible ionic liquids loaded micro/nanocapsules (Figure 8C, ii) 200. The heat generation mechanism of ionic liquids may be associated with MW electromagnetism-induced ion movement, molecular arrangement, and charged ion shift 121, 201-203. Under MW irradiation, the microcapsular structure could be decomposed owing to the increased temperature, leading to the active release of drugs 200. However, current MWA requires the cooperation of percutaneous puncture technology, and non-invasive methods are still not practicable for treating large and deep tumors due to energy dissipation.

#### 2.5.4 Photothermal sensitive embolic microspheres

Photothermal therapy (PTT) has attracted extensive attention due to its unique advantages including minimal invasiveness, and high specificity, which achieves tumoricidal effects *via* converting light energy (a high-frequency electromagnetic radiation) into heat. However, the current PTT is restricted by the limitations of low tissue penetration, since the laser rapidly attenuates with increasing tissue depth. Although researchers have explored near-infrared (NIR) laser (650 - 950 nm) with high physiological transmissivity and photothermal-sensitive micro/nano materials with enhanced photothermal conversion efficiency, interventional PTT is still needed for deep-seated tumors. For example, laser-induced interstitial thermotherapy (LITT) that utilizes flexible optical fibers to generate cytotoxic temperature within deeply buried tumors, is frequently used after TACE to improve the therapeutic effect of large-sized HCC. Like other combination therapy, the TACE plus LITT also showed better tumor regression 175 and significantly longer overall survival than monotherapy of LITT 204 or TACE 205.

In order to further improve the therapeutic effect and simplify the repeated embolization step, the development of photothermal-sensitive embolic microspheres is meaningful. The basic strategy is similar to other stimulation-susceptible embolic agents introduced earlier, that is, the inclusion of photothermal conversion agents (such as gold 125, iron oxide 206, and bismuth 124, 207 based nanoparticles) into the embolic matrix (Figure 8D). For example, Huang et al. synthesized photothermal-sensitive composite microspheres by an inverse emulsion copolymerization, in which polydopamine coated SPIO NPs (SPION@PDA) and doxorubicin were encapsulated (Figure 8D, i) 206. Although satisfactory tumor responses (tumor size decreased by 91.5 %) were achieved with the combination treatment of TACE plus PTT in VX2 liver tumor-bearing rabbits, the supplemental therapeutic mechanism of PTT still required laparotomy to expose the hepatic tumor, which is a bit superfluous. In short, compared to other non-invasive hyperthermia modalities, pure material innovation is only the “icing on the cake” for the combination therapy of TACE plus PTT, while there is still a long way to go.

#### 2.5.5 Radiofrequency sensitive embolic agents

Radiofrequency ablation (RFA) as a valuable treatment for unresectable HCC 178 has been widely considered as the gold standard therapy treatment in combination with TAE/TACE 179. During the RFA, the friction heat can be generated in targeted tumor sites *via* ionic agitation, owing to the high-frequency alternating currents launched from needle-electrodes (directly inserted into tumor nodules) 208. However, the RFA of irregularly shaped tumors is still challenging since the friction heat losses rapidly in the hypervascular tumor regions. Similarly, TAE/TACE has been used synergistically with RFA to reduce the blood flow “cooling” effect in the ablation zone, and superior therapeutic outcomes were found in clinical practice 209, 210.

Recently, several radiofrequency (RF) sensitive embolic agents have been applied to coordinate the antitumor efficacy of TAE/TACE and RFA 183, 211. For example, Li et al. synthesized RF-sensitive embolic agents (dvGC@PNAs), in which the temperature-sensitive poly (N-isopropylamide-co-acrylic acid) (PNAs) was modified onto RF-sensitive dual-valent gold nanoclusters (dvGC) *via* gold-sulfur coordination bond (Figure 8E, i) 183. Vascular embolization can be achieved when the dvGC@PNAs were infused into the tumor arteries, attributing to temperature-sensitive sol-gel transition of PNAs (maintain flowability at room temperature and convert to high gelation strength at body temperature). When RFA therapy was administered at 3 d post-TAE operation, the dvGC@PNAs mediated the synergistic effect of TAE and RFA was realized due to the RF-induced heating effect of dvGC. More importantly, they substantiated that the tumor microenvironment post-TAE procedure was greatly improved, ascribing to a favorable immune response induced by the RF-responsive dvGC@PNAs. In their subsequent research, the cisplatin-crosslinking PNAs nanogels (Pt-PNAs) were further developed as RF-responsive embolic nano-platform *via* the coordination bonding between Pt (II) ions and carboxyl (Figure 8E, ii), which could be used for improving the synergistic effect of TACE and RFA 211. In the future, it may be possible to realize a more efficient combination therapy of TAE/TACE and RFA, relying on RF-responsive material innovation. However, the invasive treatment model of RFA remains an insurmountable challenge.

## 3. Multifunctional fully flexible embolic agents

As previously mentioned, the performance of embolic microspheres is decisive for the improvement of TACE. Currently, the fundamental embolic function and drug-loading/releasing properties are associated with the embolic matrix, while the enhanced functionalities are derived from the additional components. However, simply increasing the variety of additives with beneficial functions will undoubtedly increase the complexity of the embolic system. In addition, most inclusions are rigid and insoluble inorganic materials, that generally lack surface modification (especially those formed *in situ*). Once the encapsulated microspheres are broken or degraded, the naked rigid inclusions will be released and exposed to the complex ionic microenvironment, causing hidden dangers in systemic circulation. Specifically, there may be less intracellular uptake due to the lack of receptor-ligand interaction 212, 213 and more aggregation due to high surface energy 214, 215, leading to potential adverse effects (e.g., prolonged circulation and metabolism, and increased potential for complications). Therefore, gallium (Ga) based liquid metals (LMs) with amorphous properties (superb fluidity, shape transformability, excellent flexibility, low viscosity, and self-healing capability) and inherently diverse functions (good biocompatibility, biodegradability, and facile functionalization accessibility) have aroused widespread interest in the TACE 216-219.

**Figure 9:**
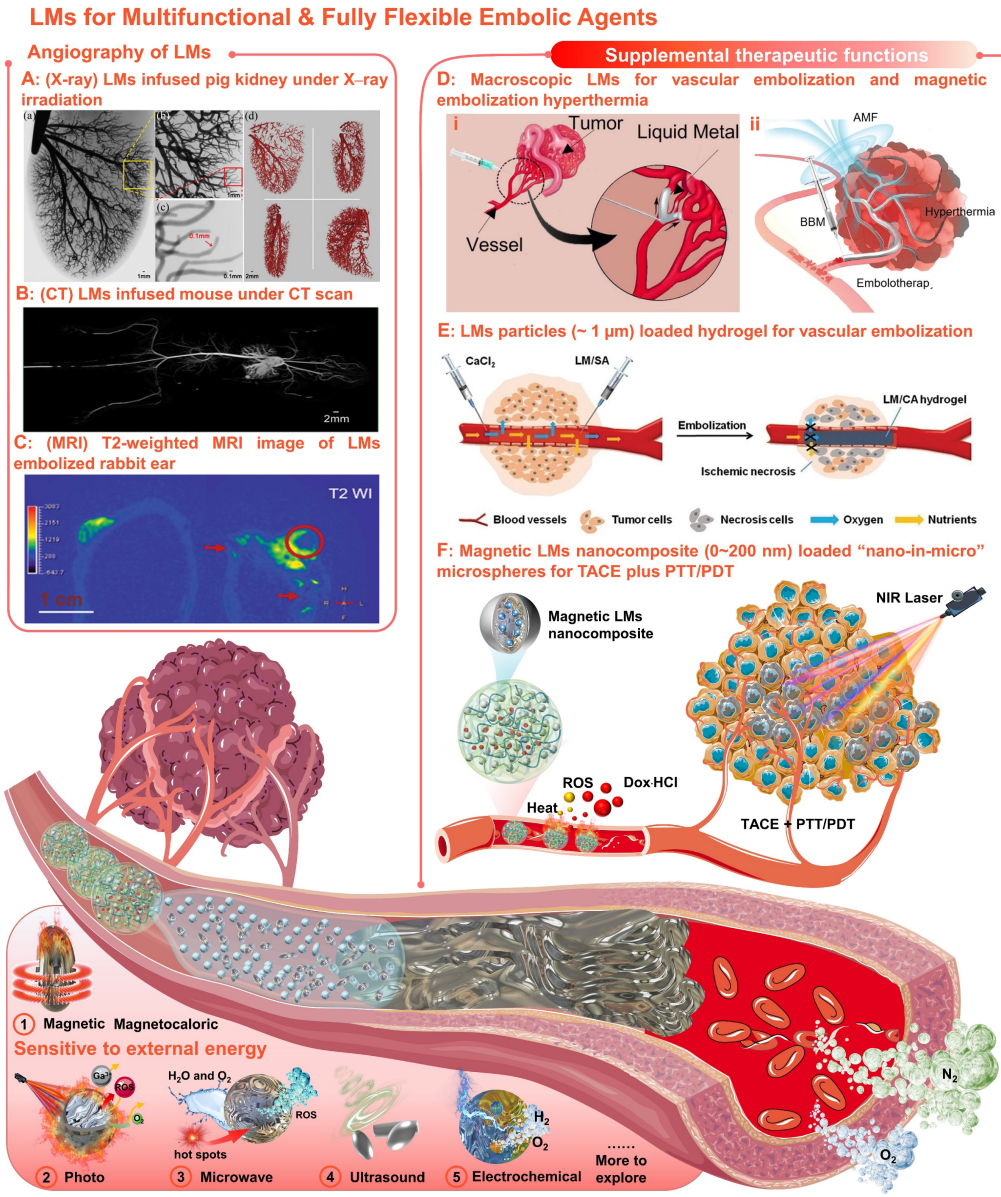
** With inherent softness, high density, electromagnetic properties, and sensitivities to external energy fields, LMs could be used as multifunctional & fully flexible embolic agents with no size limitations or redundant additives requirements.** (A) Mega contrast vasculature of a pig kidney infused with LMs (Ga) under X-ray irradiation. Adapted with permission from 220, Copyright 2014 IEEE-INST. (B) The CT images of mouse vessels filled with LMs (Ga). Adapted with permission from 221, Copyright 2014 Wang et al. (C) T_2_-weighted MRI image of (left) the tumor-bearing rabbit ear and (right) the LMs embolized rabbit ear; The red circle indicated the embolized tumors and the red arrows indicated vessels embolized with LMs (mixture of Bi_35_In_48.6_Sn_15.9_Zn_0.4_ and Ga_67_In_20.5_Sn_12.5_). Adapted with permission from 223, Copyright 2022 Wiley-VCH. (D) Macroscopic LMs for (i) vascular embolization (adapted with permission from 221, Copyright 2014 Wang et al. and (ii) magnetic embolization hyperthermia (adapted with permission from 223, Copyright 2022 Wiley-VCH). (E) The LMs particles (~ 1 µm) combined with alginate hydrogel for vascular embolization. Adapted with permission from 222, Copyright 2019 Wiley-VCH. (F) Soft magnetic LMs nanocomposite (0~200 nm) loaded “nano-in-micro” microspheres for TACE plus photothermal and photodynamic therapy (PTT/PDT). Reproduced with permission from 224, Copyright 2021 The Royal Society of Chemistry. Created in Smart.Servier.com.

Meaningfully, our united team has revealed that Ga-based LMs offer broad prospects in the field of angiography and intravascular embolization 220-224. Primary, angiography is of great significance for TACE, since vascular visualization can help to diagnose and evaluate physiological conditions related to blood vessels. With inherent softness, high density, and electromagnetic properties, the LMs can serve as effective medical imaging contrast agents for X-rays 220, CT 220, and MRI 223. Particularly, when the LMs infused into the vessels, mega contrast X-ray images (Figure 9A) 220 and CT images (Figure 9B) 221 could be generated for multiscale vasculature mapping with high radiographic densities (several orders of higher resolution than that of the Iohexol) and increased penetration depth (visualize small capillaries, ~100 µm). And the LMs also showed negative T2-weighted MRI contrast enhancement at the vascular embolism site (Figure 9C) 223. Besides, the LMs functional materials that are sensitive to external energy fields (e.g., magnetic-responsive 223, photo-responsive 225, microwave-responsive 203, ultrasonic-responsive 226, and electrochemical-responsive 227, Figure 9) could also provide supplemental therapeutic functions to TACE without the need of redundant additives. After preliminary verification of the possibility of the macroscopic LMs in vascular embolization (Figure 9D, i) 221, the macroscopic LMs as non-magnetic magnetocaloric sensitizers were also applied for magnetic embolization hyperthermia (Figure 9D, ii) 223. However, the toxicity and the degradability of the macroscopic LMs should be further evaluated systematically. After that, the LMs particles (~ 1 µm) were further combined with alginate hydrogel for vascular embolization (Figure 9E) 222. Yet, such *in situ* cross-linked LMs-gel embolic agents could only apply to superficial endovascular embolization. In order to meet clinical needs, our group first explored the soft magnetic LMs nanocomposites for the construction of “nano-in-micro” embolic microspheres, which can be used as multifunctional fully flexible embolic microspheres for dual-modality imaging guided and NIR laser enhanced TACE (Figure 9F) 224. In particular, these LMs-based microspheres were successfully employed for CT and MR dual-modality imaging, which can not only meet the X-ray radiopacity requirements of current clinical TACE, but also show potential in future MRI-guided TACE to avoid X-ray radioactive hazards. And their paramagnetism also shows potential for magnetic targetability. In addition, their photothermal and photodynamic susceptibility are beneficial for photothermal conversion, ROS generation, and controllable drug release, showing the synergistic antitumor effect of TACE and photothermal/photodynamic therapy. Most importantly, such microspheres were successfully applied for the standard TACE procedures on a domestic pig and a New Zealand white rabbit.

To sum up, different from conventional rigid micro/nano materials, inherently functional LMs will show unique compatibilities in intravascular treatment, providing a facile and versatile strategy to extend the pool of multifunctional fully flexible embolic materials. Theoretically, diverse flexible embolic materials with multiple imaging modes and supplemental therapeutic functions are readily available through the combinatorics of LMs micro-nanomaterials and matrix materials, promising for researchers to explore in the field of TACE.

## 4. Future Outlooks

As surveyed above, numerous desirable properties of particulate embolic agents have been identified and described in this review. It can be found that due to the intervention of micro/nano technology, the embolic devices have replaced complexity with simplicity while integrating more complete functionality, which will greatly simplify the TACE procedure and improve therapeutic efficiency.

**Figure 10:**
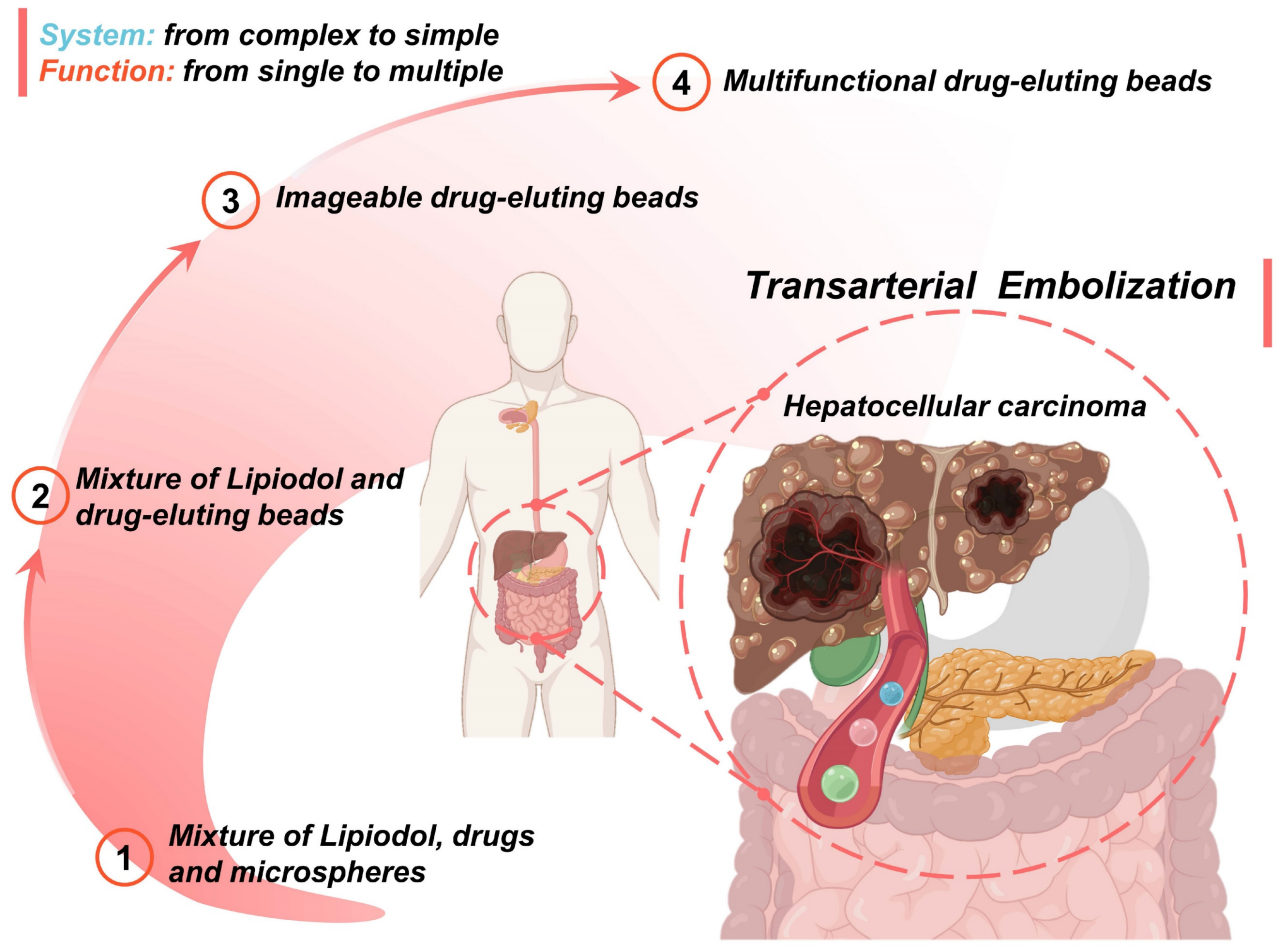
Schematic illustration of the current development routes and future trends of micro/nano embolic agents in transarterial embolization for hepatocellular carcinoma. Created with BioRender.com.

An overview of the current development route and future trends is as follows (Figure 10): 1) The first-generation TACE embolic agents: a mixture of Lipiodol, drugs, and microspheres 15, 19, 36-39; 2) The second-generation TACE embolic agents: Lipiodol and drug-eluting beads 62. The use of DEBs significantly improved the drug delivery system, which is a mature first step to incorporating drug loading and releasing functions into the clinical TACE procedures 60, 63. 3) The third-generation TACE embolic agents: imageable drug-eluting beads 38. The imageable embolic particles that are foreseeable towards clinical TACE can offer a more precise and controlled procedure than the current use of iodide contrast agents. A typical example is commercially available radiopaque drug-eluting beads (DC Bead LUMI™), which can provide inherent long-term radiopacity as well as the reliable performance of DC Bead 122. As for whether the multi-mode imageable embolic particles can be used in clinical practice, it depends on the development of medical imaging technology in the TACE procedure. 4) The new generation of TACE embolic agents: multifunctional integrated drug-eluting beads 224. Although the development of these multifunctional embolic microspheres is still in the early stages, it is undeniable that these essential properties play an essential role. For instance, stimulus sensitivity (e.g., magnetic-responsive 223, photo-responsive 225, microwave-responsive 203, ultrasonic-responsive 226, and electrochemical-responsive 227) is a reliable way to actively control drug concentration and provide adjuvant therapy. Considering the various cellular and molecular factors involved in the progression of HCC, monotherapy may not be beneficial, especially for large tumors 21. Fortunately, extensive clinical studies have supported improved outcomes of the combination therapy of TACE and various interventional thermal/nonthermal ablation modalities 14, 179-181. In the future, the combination of TACE and non-invasive hyperthermia (e.g., MIH 145, 171, MWA) 172, 173, HIFU thermotherapy 120, 170, 174, and PTT 175, 176) may become an important direction, because of inherent complementary characteristics.

Besides, these ablative modalities can also stimulate an antitumor immune response by locally releasing tumor antigens, however, tumoricidal effects are usually suppressed by the immunosuppressive tumor microenvironment of HCC (immunosuppressive mechanisms involving impaired tumor-associated antigen-processing and presentation, lack of CD4^+^ T-cell responses, enhanced myeloid-derived suppressor cells, enhanced regulatory T cells, and increased expression of programmed cell death ligand-1 21). After receiving neoadjuvant TACE, the ablative modalities in conjunction with antigen-presenting cells (e.g., dendritic cells), or cytotoxic cells (e.g., cytokine-induced killer cells), may offer a potential strategy to augment tumoricidal effects *via* counteracting the immunosuppressive mechanisms of HCC 179. Another essential function is controllable localization, considering that the current embolic microspheres localized mainly depends on the superselective insertion of the microcatheter and the size-dependent accumulation. In this regard, magnetic embolic agents have shown the preliminary possibility of controllable embolic localization 171, 224. Moreover, other noteworthy particulate embolic agents that may have an impact on future TACE treatment include LMs micro/nano materials, thrombin encapsulated micro/nano materials 228, and radioactive microspheres (for non-thermal combination therapy, e.g., TACE plus radiation Therapy 156). Furthermore, the TACE as a necrosis-inducing treatment may unmask tumor rejection of antigen-mediated immunity, thus providing a rationale for combining TACE with immunotherapy 229.

To sum up, the use of functional micro/nano materials to obtain ideal multifunctional particulate embolic agents may not be far from reality and may lead to a more comprehensive treatment method. However, for the intravascular application of these micro/nano materials, long-term research on the biodistribution, toxicity, and biocompatibility in the human body is required, and standard surgical procedures also need to be studied.

## 5. Conclusions

In summary, this review systematically identified and described recently emerging micro/nano materials as particulate embolic agents, with emphasis on materials, typical features, various functions, and practical applications. Particularly, new insights into the liquid metals-based multifunctional and flexible embolic agents were highlighted, which may have a broader impact on future angiography and intravascular embolization. Besides, the current development routes and future outlooks of these emerging micro/nano embolic materials were also concluded. Overall, the development of next-generation embolic agents with properties such as degradability, drug-loading and releasing properties, detectability, targetability, and multiple therapeutic modalities is crucial for the improvement of TACE. This review provides an in-depth understanding of newly developed micro/nano embolic agents, which may inspire multidisciplinary researchers to collaboratively innovate the next generation of embolic agents.

## Figures and Tables

**Table 1:** Summary of commercially available drug-eluting beads 18, 56, 73, 74.

Product name	DC Bead^®^	HepaSphere^®^	Tandem^®^	LifePearl^®^
**Company**	BTG, London, UK	Merit Medical, South Jordan, UT, USA	CeloNova BioSciences, Inc., San Antonio, TX, USA	Terumo European Interventional Systems, Leuven, Belgium
**Materials composition**	Acrylamido-polyvinylalcohol-AMPS hydrogel microspheres	Poly (vinyl alcohol-co-acrylic acid) microspheres	Poly (methylacrylic acid) microspheres coated with Polyzene-F	Polyethylene glycol-AMPS based microspheres
**Available size (µm)**	70-150, 100-300, 300-500, 500-700	30-60, 50-100, 100-150, 150-200	40, 75, 100, 250, 400, 500, 700, 900	100, 200, 400
**Specific properties**	Spherical, calibrated sizes, nonabsorbable, contain sulfonate binding groups	Calibrated, dry microspheres, absorbable, contain carboxylate binding groups	Spherical, calibrated sizes, nonabsorbable, contain carboxylate binding groups	Spherical, calibrated sizes, nonabsorbable, tinted green, contain sulfonate binding groups
**Drug type**	Doxorubicin and irinotecan	Doxorubicin, irinotecan, epirubicin, cisplatin or oxaliplatin	Doxorubicin and irinotecan	Doxorubicin, irinotecan, idarubicin, and epirubicin
**Drug loading efficiency (maximum doses)**	Doxorubicin (37.5 mg mL^-1^) and irinotecan (50 mg mL^-1^)	Doxorubicin (3 mg mg^-1^ of microspheres) and irinotecan (4 mg mg^-1^ of microspheres)	Doxorubicin (50 mg mL^-1^) and irinotecan (50 mg mL^-1^)	Doxorubicin (37.5 mg mL^-1^), irinotecan (50 mg mL^-1^), idarubicin (5 mg mL^-1^) and epirubicin (25 mg mL^-1^)

**Table 2:** Selection of imageable embolic microsphere systems described in the literature.

Imaging modality	Embolic matrix	Imaging component	Method of inclusion	Comments	Study (year)	Ref.
X-ray	PHEMA	Iodine (triiodobenzyl groups)	Covalent coupling	25-30 wt% loading	Horak *et al*. (1987)	94
X-ray	PMMA (hydrolyzed)	Barium sulfate	Precipitation	70 wt% loading achieved	Thanoo and Jayakrishnan (1989)	95
X-ray	PHEMA	Iodine (iothalamic/iopanoic acid)	Covalent esterification	30 wt% loading achieved	Jayakrishnan *et al*. (1990)	96
X-ray	PHEMA	Barium sulfate	Entrapment	40-50 wt% loading achieved	Thanoo and Jayakrishnan (1990)	97
X-ray	Silicone	Tantalum powder	Entrapment	Needed surface modn.	Thanoo and Jayakrishnan (1991)	98
X-ray	PHEMA copolymer	Iodine (triiodobenzyl monomer)	Copolymerization	27 wt% achieved	Horak *et al*. (1997)	99
X-ray	PHEMA/PVP copolymers	Iodine (monoiodobenzyl monomer)	Copolymerization	20 wt% achieved	van Hooy-Corstjens *et al*. (2008)	100
CT	Alginate	MoS2 nanosheets	Entrapment	12% loading	Fu *et al.* (2017)	101
CT	PLGA	Iodine (2,3,5-triiodobenzoic acid (TIBA))	Entrapment	23.15 wt % Iodine loading/Sorafenib loading demonstrated	Choi *et al.* (2017)	102
CT	Polystyrene	Tantalum oxide	Entrapment	9.4 wt% tantalum oxide loading	Morrison *et al.* (2015)	103
CT	PLAU	Iodine (4,4′-isopropylidinedi-(2,6-diiodophenol) (IBPA))	Copolymerization	14.48 wt% Iodine loading	Sang *et al.* (2017)	72
CT	PVAL	Iodine (4-iodobenzyl or 2,3,5-triiodobenzyl groups)	Copolymerization	40-70 wt % Iodine loading	Agusti *et al.* (2015)	104
MR	Trisacryl (Embosphere)	Iron oxide (SPIO)	Entrapment	Detectable by common echo sequences	Namur *et al*. (2007)	105
MR	Trisacryl (Embosphere)	Iron oxide (SPIO)	Entrapment	100% detectable	Lee *et al*. (2008)	106
MR	PVA	Gadolinium III Chelates	Covalent coupling	45.5 μg Gd(III)/mg PVA	Cilliers *et al*. (2008)	107
MR	Chitosan	Iron oxide (SPIO)	Entrapment	1.0 mM SPIO loading	Chung *et al*. (2012)	108
MR	Alginate	Prohance^®^ and Holmium ions	Complexation/entrapment	T1 MRI & T2 MRI 0~1.35wt% Ho^3+^ loading	Van *et al.* (2015)	109
MR	Alginate	Iron oxide (SPIO)	Entrapment	0.06 ~ 6.0 mg/mL SPIO loading	Wang *et al*. (2017)	67
MR/gamma	Alginate	Holmium	Complexation	1.3 wt% Ho loading	Zielhuis *et al*. (2007)	110
CBCT/MR	Alginate	Holmium and iodine (Lipiodol)	Complexation/entrapment	0.38% Ho loading	Oerlmans *et al*. (2015)	111
DSA/CT	Alginate	Barium sulfate	Complexation	Microfluidic method	Wang *et al*. (2015) Du *et al.* (2018)	88, 112
DSA/CT	Alginate	Tantalum nanoparticles	Entrapment	10 w/v% Ta loading	Zeng *et al.* (2018)	93
DSA/CT/MRI	P(MAOETIB-GMA)	Iodine/ Iron oxide (SPIO)	Copolymerization/ Precipitation	diameter 40-200 µm	Bartling *et al.* (2011)	113
Radiography/MR/CT	PMAA (Embozene)	Barium sulfate/iodine/iron oxide	Precipitation/entrapment	Three different loading densities	Stampfl *et al*. (2012)	114
Fluoro/μCT/CT	PVA-AMPS (DC/LC bead)	Iodine (Lipiodol)	Entrapment	Dose-dependent imaging	Sharma *et al*. (2010)	115
Fluoro/μCT/CT	PVA-AMPS (DC/LC bead)	Iodine (Lipiodol)	Entrapment	Correlation with drug	Dreher *et al*. (2012)	116
Fluoro/μCT/MDCT/CBCT	PVA-AMPS (DC/LC bead)	Iodine (Lipiodol)	Entrapment	Different imaging modes	Tacher *et al*. (2016)	117
μCT/CT	PVA-AMPS (DC/LC bead)	Iodine (triiodobenzyl groups)	Covalent attachment	Drug loading demonstrated	Negussie *et al*. (2015)	118
Fluoro/μCT/CT	PVA-AMPS (DC/LC bead)	Iodine (triiodobenzyl groups)	Covalent attachment	IR imaging reading study	Duran *et al*. (2016)	119
US/MR/PA	PLGA	SPIO/Perfluorohexane	Entrapment	double-emulsion process	You *et al.* (2016)	120

Note: CBCT: Cone-beam computed tomography; CT: Computed tomography; DSA: Digital subtraction angiography; Ho: Holmium; modn: Modification; MDCT: Multidetector computed tomography; MR: Magnetic resonance; PHEMA: Poly(2-hydroxyethyl methacrylate); PLAU: Poly(lactic acid)-polyurethane; PLGA: poly(lactic-co-glycolic acid); P(MAOETIB-GMA): homopolymerization of 2-methacryloy-loxyethyl (2,3,5-triiodobenzoate) (MAOETIB) with glycidyl methacrylate (GMA). PMMA: Poly(methylmethacrylate); PVP: Poly(N-vinyl-2-Pyrrolidone); PMAA: Poly(methylacrylic acid); PVA-AMPS: Poly(vinyl alcohol-co-2-acrylamido-2-methylpropane sulfonate); SPIO: Super paramagnetic iron oxide; US: Ultrasound; PA: Photoacoustic. Expanding based on the work of Lewis et al. 38

**Table 3:** Summary of commercially available imageable beads 38, 73, 74.

Feature	LC Bead LUMI^TM^	DC Bead LUMI^TM^	X-Spheres^®^
**Company**	BTG	BTG	Interface Biomaterials
**Imaging modality**	X-ray	X-ray	X-ray
**Material**	Triiodobenzyl (TIB)-modified acrylamido-polyvinylalcohol-AMPS hydrogel microspheres	TIB-modified acrylamido-polyvinylalcohol-AMPS hydrogel microspheres	TIB-modified acrylic microspheres
**Size (μm)**	70-150, 100-300	70-150, 100-300	400-600, 600-710, 710-850
**Inclusion method**	Covalently bounding of iodine moiety (TIB) into the PVA hydrogel structure	Directly coupling of TIB groups to the 1,3-diol units of the beads	Polymerization of methacrylate monomer that contains covalently bound iodine-derivative
**Time to market**	Cleared by the US FDA in December 2016	CE marked in March 2017	First authorized in 2015
**Labeled indication**	Hypervascular tumors and arteriovenous malformations (AVMs)	Nonmalignant hypervascular tumors and AVMs	

FDA: Food and Drug Administration; CE: Communate Europpene.

**Table 4:** Characteristics and differences of commercially available ^90^Y-particles 152, 156.

Feature	TheraSphere^®^	SIR-Spheres^®^
Isotope	90Y	90Y
Half-life (h)	64.2	64.2
Material	Glass	Resin
Diameter (μm)	20-30	20-60
Activity per particle (Bq)	2500	50
Spheres per 3 GBq	1.2 × 10^6^	40-80 × 10^6^
Activity in the vial (GBq)	3, 5, 7, 10, 15, or 20 %	3% ± 10%
Number of microspheres (vial, million)	1.2-8	40-80
Specific Gravity (g/mL)	3.2	1.6
Embolic effect	Negligible	Mild
Contrast injection	No	During infusion
FDA approved indication	Unresectable HCC	CRC liver metastases with intrahepatic floxuridine

FDA: Food and Drug Administration; CRC: Colorectal cancer; HCC: Hepatocellular carcinoma.
